# Medication‐related problems in older people in Catalonia: A real‐world data study

**DOI:** 10.1002/pds.5149

**Published:** 2020-11-26

**Authors:** Amelia Troncoso‐Mariño, Tomás López‐Jiménez, Albert Roso‐Llorach, Noemí Villén, Ester Amado‐Guirado, Marina Guisado‐Clavero, Sergio Fernández‐Bertolin, Mariona Pons Vigues, Quintí Foguet‐Boreu, Concepción Violán

**Affiliations:** ^1^ Àrea del Medicament i Servei de Farmàcia, Gerència Territorial de Barcelona Institut Català de la Salut Barcelona Spain; ^2^ Department of Clinical Sciences University of Barcelona and IDIBELL. L'Hospitalet de Llobregat Barcelona Spain; ^3^ Central Research Unit Fundació Institut Universitari per a la Recerca a l'Atenció Primària de Salut Jordi Gol i Gurina (IDIAPJGol) Barcelona Spain; ^4^ Departament de Pediatria, d'Obstetrícia i Ginecologia i de Medicina Preventiva Universitat Autònoma de Barcelona, Bellaterra (Cerdanyola del Vallès) Barcelona Spain; ^5^ Àrea de Serveis Assistencials Servei Català de la Salut Barcelona Spain; ^6^ Department of Psychiatry Vic University Hospital Barcelona Spain; ^7^ Department of Basic and Methodological Sciences, Faculty of Health Sciences and Welfare University of Vic‐Central University of Catalonia (UVic‐UCC) Vic Spain

**Keywords:** contraindicated drugs, drug interactions, duplicate therapy, inappropriate prescribing, multimorbidity, older people, pharmacoepidemiology

## Abstract

**Purpose:**

The aim of this study was to determine medication‐related problems (MRPs) in primary care patients over 65 years of age.

**Methods:**

Cross‐sectional study based on the electronic health records of patients (65‐99 years of age) visited in 284 primary health care centers during 2012 in Catalonia. Variables: age, sex, sociodemographic variables, number of drugs, kidney and liver function and MRPs (duplicate therapy, drug‐drug interactions, potentially inappropriate medications [PIMs] and drugs contraindicated in chronic kidney disease and in liver diseases). Unconditional logistic regression models were used to identify the factors associated with MRPs in patients with multimorbidity.

**Results:**

916 619 older people were included and 853 085 of them met the criteria for multimorbidity. Median age was 75 years and 57.7% of them were women. High percentages of MRPs were observed: PIMs (62.8%), contraindicated drugs in chronic kidney disease (12.1%), duplicate therapy (11.1%), contraindicated drugs in liver diseases (4.2%), and drug‐drug interactions (1.0%). These numbers were higher in the subgroup of patients with ≥10 diseases. The most common PIMs were connected to drugs that increase the risk of fall (66.8%), antiulcer agents without criteria for gastroprotection (40.6%), and the combination of drugs with anticholinergic effects (39.7%). In the multivariate analysis, the variables associated with all MRPs among the patients with multimorbidity were the number of drugs and the number of visits.

**Conclusions:**

The coexistence of multimorbidity and polypharmacy is associated with an elevated risk of MRPs in older people. Medication safety for older patients constitutes a pressing concern for health services.

**KEY POINTS**

1. The increase in life expectancy is associated to an increase in multimorbidity.
2. Older people belong to a population subgroup that is very prone to suffering from MRPs.
3. MPRs are very prevalent in older people with multimorbidity, mainly in the subgroup of patients with 10 or more comorbidities, and with polypharmacy.
4. There is a positive correlation between the number of drugs and the number of visits and the risk of MRPs.
5. The clinical approach in older patients with multimorbidity and the safe use of the drugs which are prescribed to them represent two main challenges for health systems in order to reach a balance between the beneficial and drug side effects.

## 1 INTRODUCTION

People over 65 years of age are the fastest‐growing segment of the world's population. It is considered that by 2050 one in four persons living in Europe and North America will be 65 or over.^1^ Increased life expectancy is associated with multimorbidity (coexistence of two or more chronic diseases), which frequently entails the prescription of multiple medications. Currently, approximately 50% of older people with multimorbidity take five or more drugs chronically. In the short to medium term, the absolute number of older people that are prescribed multiple medications is expected to grow exponentially.^2^, ^3^

Prescription of multiple medicines in older people has been associated with lack of adherence, increase in medication‐related problems (MRPs), the emergence of geriatric syndromes and diminished functional capacity, resulting in a higher consumption of health resources.^4^, ^5^ An MRP is defined as an event or circumstance involving drug therapy that actually or potentially interferes with desired health outcomes.^6^ Commonly observed MRPs in older people are lack of adherence and inappropriate prescribing.^7^ Critically, physiological changes associated with aging can alter the pharmacokinetics and pharmacodynamics of drugs.

Some studies show that multimorbidity is closely related to the emergence of MRPs, since multimorbidity often requires the intervention of various specialists and the prescription of multiple drugs.^8^ However, there is a lack of information on prescription safety in older people in relation to multimorbidity. The aim of this study was to determine the association between MRPs and multimorbidity in primary care patients over 65 years of age in Catalonia (Spain).

## 2 MATERIALS AND METHODS

### 2.1 Design, setting and study population

A cross‐sectional study was carried out in Catalonia (Spain), a Mediterranean region of 7 515 398 inhabitants.^9^ The Spanish National Health Service provides universal coverage, financed mainly by tax revenue. The Catalan Institute of Health (ICS) manages 285 primary health care centers (PHC) in Catalonia that serve 5 501 784 people,^10^ of which 16.85% were over 65 years of age.

Inclusion criteria were: individuals between 65 and 99 years old in December 31, 2011 and assigned to a PHC managed by ICS. Only participants that survived until December 31, 2012, were included in the analysis. A total of 916 619 patients were included at baseline. The study period was from January 1, 2012 to December 31, 2012.

### 2.2 Data sources

The Information System for Research in Primary Care (SIDIAP) contains anonymized clinical information originating from the primary care electronic health records (EHR) since 2006.^11^

The medication database includes drugs subsidized, dispensed and billed by the national health system. For the purpose of identifying problems related to the most relevant medications, the study only included systemic drugs, and excluded hospital medication, drugs dispensed by hospital pharmacies, drugs not subsidized by public health services and topical medication (eg, ointments and lotions).

### 2.3 Variables

All variables were obtained directly from the SIDIAP database.^12^

#### 2.3.1 Chronic diseases and multimorbidity

In the SIDIAP database, diseases are coded in accordance with the International Classification of Diseases, version 10 (ICD‐10). An operational definition of multimorbidity was used, that is, the presence of two or more chronic diseases, based on the selected 60 groups of chronic diseases determined by the Swedish National study of Aging and Care in Kungsholmen (SNAC‐K),^13^ with additional clinical, laboratory and medication‐related parameters for the assessment of certain conditions. The number of different chronic diseases per patient was classified in four categories: 0‐1 (non‐multimorbidity); 2‐4; 5‐9; ≥10 chronic diseases.

#### 2.3.2 Drugs and polypharmacy

The number of different drugs per patient was classified in five categories: 0; 1; 2‐4; 5‐9; ≥10 drugs.

Information on drug exposure was obtained from the Pharmacy Invoice Registry, which includes drugs prescribed by primary care and hospital physicians. Drugs were classified according to the Anatomical Therapeutic Chemical Classification System (ATC).^14^ The fourth and fifth levels were used to facilitate analysis and interpretation. Chronic use was considered when the person used three packages of the drug during the study period. Each drug was coded into a dichotomous variable. Polypharmacy was defined as the concurrent use of five or more drugs by the same individual.^15^

#### 2.3.3 Kidney function

Kidney function was defined by two different parameters:

- Glomerular Filtration Rate (GFR): kidney function was calculated estimating the GFR according to the MDRD‐4 IDMS10 equation.^16^ Abnormal kidney function was considered when one or more values of GFR were <60 mL/min/1.73 m^2^.
- ICD‐10 codes included in chronic kidney disease category according to SNAC‐K criteria (see Table S1).

#### 2.3.4 Liver function

Liver function was defined by two different parameters:

- Liver function values: Alkaline Phosphatase (ALP), Alanine Transaminase (ALT); and Gamma‐glutamyl Transpeptidase (GGT). Liver dysfunction was considered when at least one value was abnormal: ALP > 2 × 129 IU/L; ALT > 5 × 41 IU/L (men) or ALT > 5 × 33 IU/L (women); and GGT > 61 IU/L.^17^
- ICD‐10 codes included in chronic liver disease category according to SNAC‐K criteria (see Table S2).

#### 2.3.5 Other variables

Additional variables included in the study were socio‐demographic variables such as age at baseline (years), sex (men, women), socio‐economic status (MEDEA index; quintiles from least to most deprived)^18^ and number of total visits to PHC in 2012.

#### 2.3.6 Medication‐related problems

The following MRPs were analyzed: duplicate therapy, drug‐drug interactions, potentially inappropriate medications (PIMs) in older people (≥65 years old), contraindicated drugs in chronic kidney disease and contraindicated drugs in liver diseases. To analyze the MRPs, firstly we created the tables that contain the medicines or associations of medicines with potential safety concerns:

- Duplicate therapy: practice of prescribing two or more medications with the same pharmacological activity. Duplicate therapies considered to pose significant clinical risk by professional consensus were included. The associations of active principles with the same pharmacological action that were used jointly to achieve a synergistic effect or to adjust doses were not considered (see Table S3).
- Drug‐drug interaction: when the activity or effect of one drug is altered by the presence or the action of another. We prioritized interactions that might threaten life due to therapeutic failure or toxicity. We used the *Thesaurus des interactions médicamenteuses* from France's *Agence nationale de sécurité du médicament et des produits de santé* with the highest level of severity (contraindication).^19^ This information was contrasted with a second source of information^20^, ^21^ or by professional consensus (see Table S4).
- Contraindicated drugs in chronic kidney disease: contraindicated medications were based on a consensus recommendation of the Catalan Health Department for patients with chronic kidney disease (see Table S5).^22^
- Drugs contraindicated in liver disease: were based on the database of the Spanish College of Pharmacists (see Table S6).^23^
- PIMs in older people (≥65 years old): were considered when the associated risk of adverse events was higher than the expected clinical benefits and when there was not clear scientific evidence for a specific indication or cost‐effectiveness. We mainly used the STOPP/START criteria,^24^ complemented with Beers' criteria,^25^ PRISCUS and updated with others sources.^26^, ^27^, ^28^, ^29^, ^30^, ^31^, ^32^, ^33^ PIMs have been classified in different categories: combination of drugs with a clinically relevant anticholinergic effect according to criteria stated by Durán et al, drugs and risk of fall, drugs that affect QT interval with known risk, use of antiulcer agents without criteria for gastroprotection, other drugs not recommended for older people and patients needing gastroprotection (see Table S7).

### 2.4 Ethics approval and consent to participate

The protocol of the study was approved by the Clinical Research Ethics Committee of the Fundació Institut Universitari per a la Recerca a l'Atenció Primària de Salut Jordi Gol i Gurina (IDIAPJGol) (Protocol No: P17/080). All data were anonymized and confidentiality of EHR was guaranteed at all times in accordance with national and international law.

### 2.5 Statistical analysis

Descriptive statistics were used to summarize overall information. Categorical variables were expressed as frequencies (percentage) and continuous variables as mean (SD) or median (interquartile range, IQR). We used the Chi‐square test and Mann‐Whitney *U* test to assess differences between groups (multimorbidity groups and non‐multimorbidity group).

Prevalence of MRPs and use of PIMs were compared among groups.

Logistic regression models were fitted in order to identify the factors associated with each MRP (duplicate therapy, drug‐drug interactions, contraindicated drugs in chronic kidney disease, contraindicated drugs in liver disease and PIMs) in the population with multimorbidity. Odd ratios (OR) and 95% confidence intervals (CI) were calculated. Variables included in the logistic regression models were sex, age, MEDEA index, number of drugs and number of visits.

The analyses were carried out using SPSS for Windows, version 25 (SPSS Inc., Chicago, IL), Stata 15 Stata/MP, version 15 for Windows (Stata Corp. LP, College Station, TX) and R version 3.6.1 (R Foundation for Statistical Computing, Vienna, Austria). The significance level was set at .05.

## 3 RESULTS

A total of 916 619 patients were included in the analysis. The median age was 75 years (IQR: 69‐81) and 57.7% were women. Out of 853 085 patients, 93.1% met multimorbidity criteria, with a higher prevalence in women (93.8% vs 92.1%, *P* < .001). In the multimorbidity group, the mean number of diagnoses per patient was 6.3 (SD: 3.0) and the mean number of medications was 5.6 (SD: 3.9). Table 1 shows the baseline characteristics of the study population.

**TABLE 1:** Descriptive characteristics of patients without multimorbidity and in the various multimorbidity groups (916 619 older people, Catalonia, 2012)

	Non‐multimorbidity (n = 63 534)	Multimorbidity (2‐4 diseases) (n = 268 836)	Multimorbidity (5‐9 diseases) (n = 463 709)	Multimorbidity (≥10 diseases) (n = 120 540)	All (N = 916 619)
Sex, women, n (%)	32 837 (51.68)	142 427 (52.98)	274 575 (59.21)	79 292 (65.78)	529 131 (57.73)
Age, median [IQR]	71 [67‐78]	72 [68‐79]	75 [70‐81]	78 [73‐83]	75 [69‐81]
MEDEA Index,^a^ n (%)					
Rural	13 059 (21.96)	58 325 (22.87)	91 325 (21.20)	19 540 (18.36)	182 249 (21.40)
Q1	13 897 (23.37)	47 821 (18.76)	67 609 (15.70)	15 464 (14.53)	144 791 (17.00)
Q2	9894 (16.64)	41 815 (16.40)	68 563 (15.92)	16 159 (15.18)	136 431 (16.02)
Q3	8976 (15.10)	40 866 (16.03)	70 941 (16.47)	17 439 (16.39)	138 222 (16.23)
Q4	7666 (12.89)	37 007 (14.51)	69 998 (16.25)	18 317 (17.21)	132 988 (15.62)
Q5	5967 (10.04)	29 139 (11.43)	62 269 (14.46)	19 508 (18.33)	116 883 (13.73)
Number of drugs, n (%)					
0	40 941 (64.44)	45 992 (17.11)	23 727 (5.12)	3577 (2.97)	114 237 (12.46)
1	8239 (12.97)	32 429 (12.06)	15 654 (3.38)	641 (0.53)	56 963 (6.21)
2‐4	11 496 (18.09)	115 655 (43.02)	119 778 (25.83)	7932 (6.58)	254 861 (27.80)
5‐9	2703 (4.25)	68 894 (25.63)	235 384 (50.76)	50 330 (41.75)	357 311 (38.98)
≥10	155 (0.24)	5866 (2.18)	69 166 (14.92)	58 060 (48.17)	133 247 (14.54)
Number of visits, n (%)					
0	23 402 (36.83)	15 814 (5.88)	7522 (1.62)	1207 (1.00)	47 945 (5.23)
1	9603 (15.11)	15 991 (5.95)	7609 (1.64)	681 (0.56)	33 884 (3.70)
2‐4	16 241 (25.56)	63 295 (23.54)	46 966 (10.13)	3937 (3.27)	130 439 (14.23)
5‐9	10 168 (16.00)	93 326 (34.71)	129 577 (27.94)	16 278 (13.50)	249 349 (27.20)
≥10	4120 (6.48)	80 410 (29.91)	272 035 (58.67)	98 437 (81.66)	455 002 (49.64)
Chronic kidney disease, n (%)	249 (0.39)	15 619 (5.81)	90 365 (19.49)	47 523 (39.43)	153 756 (16.77)
Chronic liver disease, n (%)	810 (1.27)	11 261 (4.19)	33 438 (7.21)	14 138 (11.73)	59 647 (6.51)

*Note:* All variables of the table showed a significant difference (*P* < .001) between groups.

^a^ MEDEA is a deprivation index measured in quintiles (Q), from Q1 (least deprived) to Q5 (most deprived). Missing values n = 65 055.

The most frequent MRP was PIM (62.8%), followed by contraindicated drugs in chronic kidney disease (12.1%), and duplicate therapy (11.1%). These percentages were higher in the subgroup of patients with 10 or more comorbidities, with percentages of MRP of 88.0%, 31.3% and 20.1%, respectively. Conversely, these percentages were less in non‐multimorbidity group: 16.1%, 0.06% and 1.86%, respectively (Table 2).

**TABLE 2:** Medication‐related problems by multimorbidity or non‐multimorbidity group

Medication‐related problems by multimorbidity or non‐multimorbidity group (916 619 older people, Catalonia, 2012)					
	Non‐multimorbidity (n = 63 534)	Multimorbidity (2‐4 diseases) (n = 268 836)	Multimorbidity (5‐9 diseases) (n = 463 709)	Multimorbidity (≥10 diseases) (n = 120 540)	All (N = 916 619)
Medication‐related problems				
Duplicate therapy, n (%)	1184 (1.86)	18 120 (6.74)	58 548 (12.63)	24 231 (20.10)	102 083 (11.14)
Drug‐drug interactions, n (%)	72 (0.11)	1112 (0.41)	4772 (1.03)	3073 (2.55)	9029 (0.99)
Contraindicated drugs in chronic kidney disease, n (%)	36 (0.06)	8348 (3.11)	64 466 (13.90)	37 710 (31.28)	110 560 (12.06)
Contraindicated drugs in liver disease, n (%)	178 (0.28)	5548 (2.06)	21 714 (4.68)	10 643 (8.83)	38 083 (4.15)
Potentially inappropriate medication, n (%)	10 247 (16.13)	124 847 (46.44)	333 922 (72.01)	106 120 (88.04)	575 136 (62.75)

Potentially inappropriate medications by multimorbidity or non‐multimorbidity group (575 136 older people, Catalonia, 2012)					
	Non‐multimorbidity (n = 10 247)	Multimorbidity (2‐4 diseases) (n = 124 847)	Multimorbidity (5‐9 diseases) (n = 333 922)	Multimorbidity (≥10 diseases) (n = 106 120)	All (N = 575 136)
Patients taking drugs with anticholinergic effect‐anticholinergic load^a^					
Score = 1	2009 (19.61)	22 841 (18.30)	70 393 (21.08)	26 846 (25.30)	122 089 (21.23)
Score = 2	866 (8.45)	11 850 (9.49)	42 212 (12.64)	17 483 (16.47)	72 411 (12.59)
Score = 3‐5	284 (2.77)	3939 (3.16)	18 237 (5.46)	10 426 (9.82)	32 886 (5.72)
Score ≥ 6	6 (0.06)	116 (0.09)	592 (0.18)	452 (0.43)	1166 (0.20)
Patients taking drugs and risk of fall	6505 (63.48)	77 738 (62.27)	221 337 (66.28)	78 756 (74.21)	384 336 (66.83)
Patients taking drugs that affect QT interval	1291 (12.60)	16 971 (13.59)	62 146 (18.61)	28 477 (26.83)	108 885 (18.93)
Patients taking antiulcer agents without criteria for gastroprotection	2928 (28.57)	41 150 (32.96)	137 713 (41.24)	51 485 (48.52)	233 276 (40.56)
Other drugs not recommended for older people	2078 (20.28)	36 840 (29.51)	129 781 (38.87)	53 002 (49.95)	221 701 (38.55)
Patients needing gastroprotection	25 (0.24)	341 (0.27)	886 (0.27)	222 (0.21)	1474 (0.26)

*Note:* All variables of the table showed a significant difference (*P* < .001) between groups.

^a^ Score = 0, not shown.

The most common PIMs were drugs that increase the risk of fall (66.8%), the use of antiulcer agents without criteria for gastroprotection (40.6%), and the combination of drugs with anticholinergic effect (39.7%), for which 34 052 patients (5.9%) presented high anticholinergic load (score ≥ 3). These percentages are higher in the subgroup of patients with ≥10 comorbidities (see Table 2 for more details).

**FIGURE 1:**
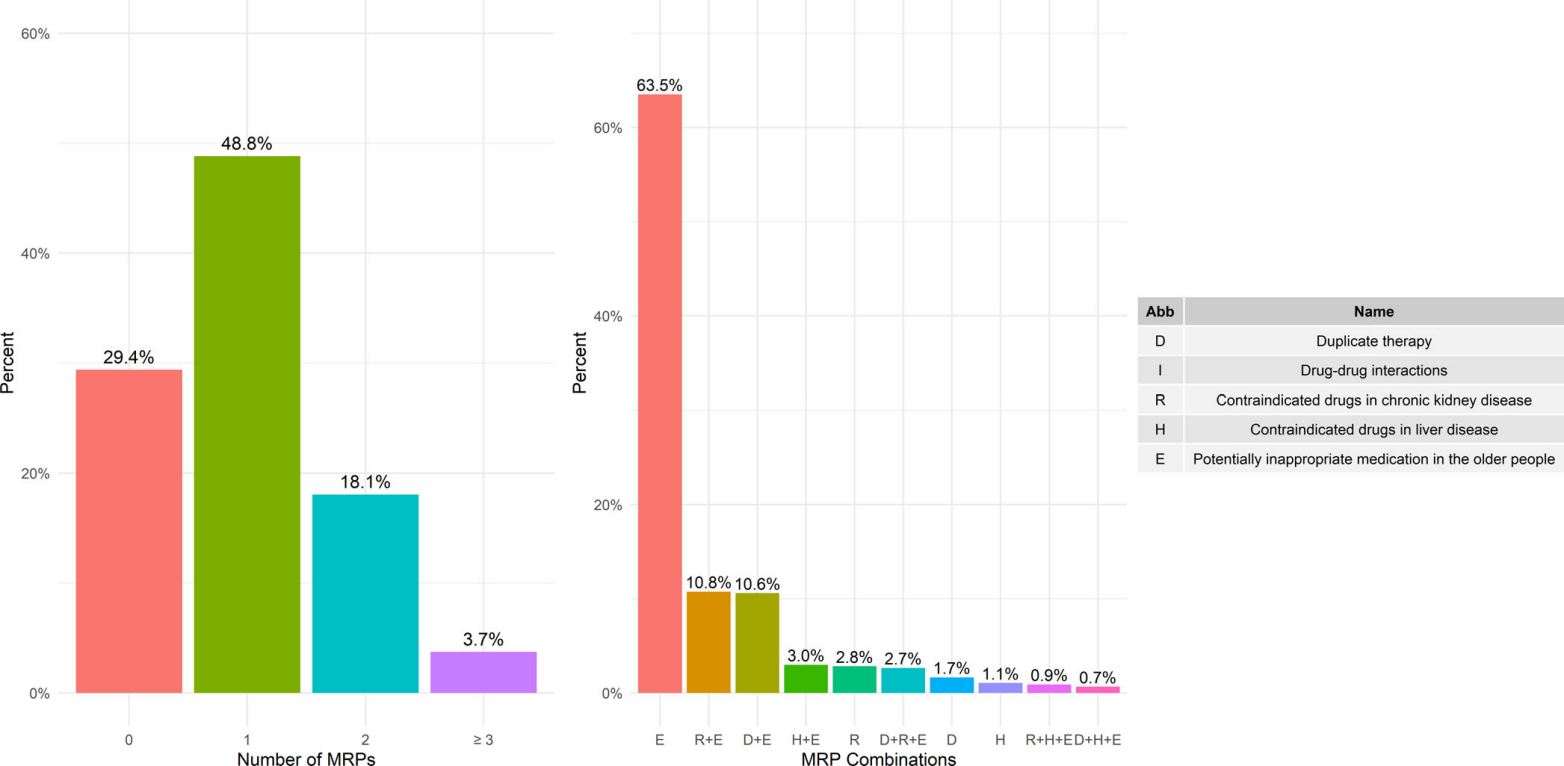
Frequencies and types of medication‐related problems in older people with multimorbidity

Only 29.4% patients with multimorbidity were free from MRPs. Conversely, 21.8% presented a combination of two or more MRPs. The most common combination was contraindicated drugs in chronic kidney disease and PIMs (10.8%), closely followed by duplicate therapy and PIMs (10.6%) (Figure 1).

A 6.7% of patients with multimorbidity and contraindicated drug in chronic kidney disease and a 2.4% of patients with multimorbidity and contraindicated drug in liver disease had two or more contraindicated medication. A 16.2% of patients with multimorbidity who suffered from a PIM‐type MRP had four or more PIMs. These percentages are higher in the subgroup of patients with ≥10 comorbidities (see Table S8).

The most common contraindicated drugs in cases of chronic kidney disease were metformin, hydrochlorothiazide, plain or in combination with enalapril, and citalopram; in liver failure, simvastatin, metformin and furosemide. The most common PIMs were the use of omeprazole without criteria for gastroprotection, the use of lorazepam, tamsulosin, lormetazepam and alprazolam because of the increased risk of fall, citalopram because of the increased risk of QT interval alteration and anti‐inflammatories (ibuprofen) (see Tables S9‐S11).

In the multivariate analysis, women showed a higher risk of MRPs, with OR that varied from 1.12 (95% CI: 1.10‐1.14) to 1.24 (95% CI: 1.19‐1.30), except in the case of drugs contraindicated in liver diseases (OR: 0.6; 95% CI: 0.58‐0.61). There was a positive correlation between the number of drugs and the risk of MRPs, particularly between PIMs and the group of patients that were prescribed ≥10 drugs (OR: 52.68; 95% CI: 50.75‐54.69). Finally, a higher number of visits (number of visits from 1 to ≥10) correlated with a higher risk of MRPs, with OR ranging from 2.70 (95% CI: 2.30‐3.17) to 23.74 (95% CI: 16.60‐33.96) (Table 3).

**TABLE 3:** Odds of each medication‐related problems according to clinical and sociodemographic variables in older people with multimorbidity (N = 853 085)

	Duplicate drugs	Drug‐drug interactions	Contraindicated drugs in chronic kidney disease	Contraindicated drugs in liver disease	Potentially inappropriate medication
Sex (women)	1.12 (1.10–1.14)	1.24 (1.19–1.30)	1.18 (1.16‐1.20)	0.60 (0.58‐0.61)	1.22 (1.21‐1.23)
Age (years)	0.98 (0.98‐0.98)	1.00 (1.00‐1.01)	1.06 (1.05‐1.06)	0.97 (0.97‐0.97)	1.01 (1.01‐1.02)
MEDEA index^a^					
Q1	1.00	1.00	1.00	1.00	1.00
Q2	0.94 (0.92‐0.97)	0.91 (0.84‐0.98)	0.96 (0.94‐0.99)	1.08 (1.04‐1.12)	0.96 (0.95‐0.98)
Q3	0.94 (0.92‐0.96)	0.89 (0.82‐0.96)	0.95 (0.93‐0.97)	1.01 (0.97‐1.05)	0.96 (0.94‐0.97)
Q4	0.88 (0.85‐0.90)	0.81 (0.75‐0.88)	0.94 (0.92‐0.96)	1.04 (1.00‐1.08)	0.92 (0.90‐0.93)
Q5	0.84 (0.82‐0.86)	0.71 (0.66‐0.78)	1.03 (1.00‐1.05)	1.07 (1.03‐1.11)	0.91 (0.89‐0.92)
Rural	0.95 (0.93–0.97)	1.08 (1.00‐1.16)	0.82 (0.80‐0.84)	1.09 (1.05‐1.13)	1.02 (1.00‐1.04)
Drugs number					
0‐4 drugs	1.00	1.00	1.00	1.00	1.00
5‐9 drugs	5.53 (5.40‐5.67)	8.20 (7.35‐9.15)	2.51 (2.46‐2.55)	2.12 (2.06‐2.19)	8.01 (7.91‐8.11)
≥10 drugs	16.95 (16.52‐17.39)	34.83 (31.19‐38.89)	4.67 (4.57‐4.77)	3.53 (3.41‐3.64)	52.68 (50.75‐54.69)
Number of visits					
0	1.00	1.00	1.00	1.00	1.00
1	2.70 (2.30–3.17)	3.07 (1.71‐5.51)	2.81 (2.34‐3.38)	4.15 (2.81‐6.12)	5.00 (4.73‐5.29)
2‐4	3.46 (2.98‐4.01)	3.05 (1.76‐5.29)	7.02 (5.95‐8.27)	9.69 (6.76‐13.89)	7.08 (6.74‐7.44)
5‐9	4.19 (3.62‐4.85)	3.02 (1.75‐5.23)	11.40 (9.69‐13.42)	15.97 (11.16‐22.84)	8.23 (7.83‐8.64)
≥10	4.75 (4.10‐5.49)	3.16 (1.83‐5.46)	13.80 (11.73‐16.24)	23.74 (16.60‐33.96)	9.35 (8.91‐9.82)

^a^ MEDEA index is a deprivation index measured in quintiles (Q), from Q1 (least deprived) to Q5 (most deprived). Missing values n = 60 980.

## 4 DISCUSSION

MRPs, specifically PIMs, are very prevalent in older people with multimorbidity and polypharmacy. Moreover, the frequency of MRPs and PIMs increase significantly with the number of comorbidities and medicines taken by the patient.

Multimorbidity is associated with polypharmacy, and the addition of medicines can result in duplicate therapy, drug‐drug interactions and the prescription of not recommended drugs. Crucially, in this cross‐sectional study we have observed that multiple drugs can contribute to the deterioration of the already fragile kidney and liver function of older people. Consequently, monitoring kidney and liver function in this population group is recommended.^34^

The result of this study with an 11.1% of patients affected by duplicated drugs is higher than the 2.5% of hospitalized Italian patients and the 4.1% of Indian patients; but lower than the 39% obtained in an American primary care study.^35^, ^36^, ^37^ In contrast, our results showed a low percentage of drug‐drug interactions, probably due to the fact that we only included drugs that were explicitly contraindicated, and missed less relevant interactions, dietary supplements and other medications that do not require medical prescription.^38^, ^39^ The likelihood of a drug‐drug interaction increase with the number of medicines, and studies indicate that it might reach up to 50% when taking 5 to 9 medications, as verified by the higher number of drug‐drug interactions in patients taking ≥10 drugs.^4^

With regard to the impact of medication on kidney function, we should underscore that measuring kidney function is complex. Our percentage of older people with multimorbidity who take drugs that are contraindicated in chronic kidney disease concurs with the results of other studies. However, it is not possible to draw a direct comparison since these other studies used different criteria.^40^, ^41^, ^42^ In this study, the inclusion of a comprehensive list of commonly prescribed drugs in primary care might have influenced the high number of patients taking contraindicated drugs in chronic kidney disease. Alarmingly, almost 12.1% of patients with multimorbidity take drugs that are contraindicated in chronic kidney disease; and, in the case of people with ≥10 diseases, the contraindicated drugs in chronic kidney disease are 10 times more prevalent than in the multimorbidity group with two to four diseases. We strongly recommend strengthening automated warnings in the EHR to reduce these percentages.

There is a lack of data on medication management regarding liver disease in older primary care people. In hospital‐based studies, the prevalence of contraindicated drugs in liver disease ranges from 8.5% to 30.6%.^43^, ^44^

A systematic review on PIMs showed a wide range of results (11.5%‐62.5%), probably attributable to the different criteria used in the various studies.^45^ Interestingly, our study presented an even higher prevalence (62.8%) of PIMs. Number of medications, advanced age and female sex are the factors more associated with prescribing inappropriate medications.^45^ A recent systematic review showed that PIMs included anxiolytics, antidepressants and nonsteroidal anti‐inflammatory drugs and anti‐rheumatic products.^46^ Similarly, we observed that anxiolytics (lorazepam and alprazolam), hypnotics (lormetazepam), antidepressants (citalopram and paroxetine) and anti‐inflammatories (ibuprofen) were the most commonly found PIMs in our study. Worryingly, drugs that increase the risk of fall and combinations of drugs with an anticholinergic effect are very commonly prescribed older people. Recent studies emphasize the risk of anticholinergic drugs in older people and the increased risk (up to 10%) of dementia associated with this type of medication.^47^

The major strength of this study is the use of a high quality database of primary care records that includes 75% of the Catalan population and is, thus, considered highly representative. This study comprehensively analyses the most commonly prescribed medicines in primary care and provides information on MRPs relating to the use of contraindicated drugs in liver diseases in older people. On the other hand, our analysis excludes hospital medications, drugs dispensed from hospital pharmacies, drugs not subsidized by public health services and medication prescribed for acute conditions, which have the potential to cause MRPs. Moreover, the study uses the billing database for drugs, while actual adherence to medication is not known.

In this study, the medication registered in the EHR includes drugs prescribed by family doctors and other specialists. Fragmentation of care is common in patients with multimorbidity, which are frequently referred to specialists, thus increasing the risk of MRPs.^48^ Family doctors apply a more holistic approach to care, while specialists tend to focus on guidelines for a specific disease.^48^ Additionally, family doctors have more opportunities to review and withdraw unnecessary medications in coordination with other specialists, and thus play a pivotal role in medication reconciliation. Non‐medical professionals such as pharmacists and nurses can also make important contributions toward this objective.^49^

There is much diversity among studies which quantify the impact of interventions aimed at reducing MRPs and PIMs. Consequently, there is unclear evidence showing that interventions such as the review of prescriptions actually reduced the number of patients with one or more PIMs or MRPs.^50^

Importantly, reduction of MRPs should be assisted by the integration of automated warnings regarding prescriptions and a safe use of medications in the prescription modules of family doctors. The results of this study indicate that the coexistence of multimorbidity and polypharmacy is clinically relevant since it correlates with an elevated risk of MRPs. However, the study cannot specify the individual impact of each of these two factors. Polypharmacy and unsafe medication constitute a risk factor for undesired clinical consequences: falls, fall‐associated variables (dizziness, fear of falling, fractures), adverse drug reactions, general health decline (weight loss, daily life activities), poor cognitive performance, hospitalization and mortality rates.^51^ However, multimorbidity has also been involved in many of these adverse events. Finally, these results correspond to a specific point in time. Further studies should address the impact of MRPs when patients become older and multimorbidity increases, and also the impact of MRPs on patients' health and on the health services.

## 5 CONCLUSIONS

The results of this study underscore the clinical significance of polypharmacy and multimorbidity, which correlate with a high risk of MRPs in patients over 65 years of age. The risk of MRPs could be attenuated with periodic reviews of medication and the implementation of automated warnings in the electronic prescription systems although the clinical impact of this reduction is not known. The inappropriately prescribed drugs most commonly related to safety issues were proton pump inhibitors and anxiolytics. With the aging of the population, ensuring safe and effective pharmacological treatment for older patients represents one of the main challenges for health services.

## CONFLICT OF INTEREST

All authors have completed the ICMJE uniform disclosure form at www.icmje.org/coi_disclosure.pdf and declare: no support from any organization for the submitted work; no financial relationships with any organizations that might have an interest in the submitted work in the previous 3 years; no other relationships or activities that could have influenced the submitted work.

## AUTHOR CONTRIBUTIONS

All authors contributed to the design of the study, revised the article and approved the final version. Concepción Violán, Albert Roso‐Llorach, Ester Amado‐Guirado and Sergio Fernández‐Bertolin obtained the funding. Concepción Violán, Mariona Pons Vigues, Quintí Foguet‐Boreu, Albert Roso‐Llorach, Amelia Troncoso‐Mariño and Noemí Villén drafted the article. Noemí Villén, Amelia Troncoso‐Mariño, Mariona Pons Vigues, Albert Roso‐Llorach, Concepción Violán and Sergio Fernández‐Bertolin proposed the methodology, conducted the tests and contributed to the analysis and interpretation of data. Amelia Troncoso‐Mariño, Concepción Violán, Quintí Foguet‐Boreu and Noemí Villén wrote the first draft of the manuscript. Noemí Villén, Amelia Troncoso‐Mariño, Albert Roso‐Llorach and Sergio Fernández‐Bertolin wrote the first draft of the supplementary file, and all authors contributed ideas, interpreted the findings and reviewed rough drafts of the manuscript.

## ETHICS STATEMENT

The protocol of the study was approved by the Clinical Research Ethics Committee, Fundació Institut Universitari per a la Recerca a l'Atenció Primària de Salut Jordi Gol i Gurina (IDIAPJGol) (Protocol No: P17/080). All data were anonymized in agreement with national and international law.

## Supporting information


**Table S1** Codes ICD 10 included in the diagnosis of chronic kidney disease.
**Table S2.** Codes ICD 10 included in the diagnosis of chronic liver disease.
**Table S3.** List of duplicate therapy.
**Table S4.** List of drug‐drug interaction.
**Table S5.** List of drugs contraindicated in chronic kidney disease by first level of ATC.
**Table S6.** List of drugs contraindicated in liver diseases by first level of ATC.
**Table S7.** List of drugs potentially inappropriate in the elderly (≥65 years).
**Table S8.** Number of contraindicated drugs in chronic kidney disease and liver disease and potentially inappropriate medications in older people by multimorbidity group (853 085 older people, Catalonia, 2012).
**Table S9.** Twenty most contraindicated drugs in chronic kidney disease by multimorbidity group (853 085 older people, Catalonia, 2012).
**Table S10.** Twenty most contraindicated drugs in liver disease by multimorbidity group (853 085 older people, Catalonia, 2012).
**Table S11.** Twenty most potentially inappropriate medications in older people by multimorbidity group (853 085 older people, Catalonia, 2012).

## Data Availability

The datasets are not available, since researchers signed an agreement with the Information System for the Development of Research in Primary Care (SIDIAP) concerning confidentiality and security of the dataset, which forbids providing data to third parties. The SIDIAP is subject to periodic audits.
