# Supplementary material for: Medication‐related problems in older people in Catalonia: A real‐world data study
Source: Pharmacoepidemiol Drug Saf. 2020 Nov 26;30(2):220–8. doi: 10.1002/pds.5149 (PMC7839740; doi:10.1002/pds.5149)
Supplement: Supplementary file 1 — Table S1 Codes ICD 10 included in the diagnosis of chronic kidney disease. Table S2. Codes ICD 10 included in the diagnosis of chronic liver disease. Table S3. List of duplicate therapy. Table S4. List of drug‐drug interaction. Table S5. List of drugs contraindicated in chronic kidney disease by first level of ATC. Table S6. List of drugs contraindicated in liver diseases by first level of ATC. Table S7. List of drugs potentially inappropriate in the elderly (≥65 years). Table S8. Number of contraindicated drugs in chronic kidney disease and liver disease and potentially inappropriate medications in older people by multimorbidity group (853 085 older people, Catalonia, 2012). Table S9. Twenty most contraindicated drugs in chronic kidney disease by multimorbidity group (853 085 older people, Catalonia, 2012). Table S10. Twenty most contraindicated drugs in liver disease by multimorbidity group (853 085 older people, Catalonia, 2012). Table S11. Twenty most potentially inappropriate medications in older people by multimorbidity group (853 085 older people, Catalonia, 2012). [file PDS-30-220-s001.docx]

**Table S1. Codes ICD 10 included in the diagnosis of Chronic kidney disease.**

| **ICD 10** | **Label** |
| --- | --- |
| I120 | Hypertensive renal disease with renal failure |
| I130 | Hypertensive heart and renal disease with (congestive) heart failure |
| I131 | Hypertensive heart and renal disease with renal failure |
| I132 | Hypertensive heart and renal disease with both (congestive) heart failure and renal failure |
| I139 | Hypertensive heart and renal disease, unspecified |
| N01 | Rapidly progressive nephritic syndrome |
| N03 | Chronic nephritic syndrome |
| N04 | Nephrotic syndrome |
| N05 | Unspecified nephritic syndrome |
| N07 | Hereditary nephropathy, not elsewhere classified |
| N08 | Glomerular disorders in diseases classified elsewhere |
| N11 | Chronic tubulo-interstitial nephritis |
| N183 | Chronic kidney disease, stage 3 |
| N184 | Chronic kidney disease, stage 4 |
| N185 | Chronic kidney disease, stage 5 |
| N189 | Chronic kidney disease, unspecified |
| Q60 | Renal agenesis and other reduction defects of kidney |
| Q611 | Polycystic kidney, autosomal recessive |
| Q612 | Polycystic kidney, autosomal dominant |
| Q613 | Polycystic kidney, unspecified |
| Q614 | Renal dysplasia |
| Q615 | Medullary cystic kidney |
| Q618 | Other cystic kidney diseases |
| Q619 | Cystic kidney disease, unspecified |
| Z905 | Acquired absence of kidney |
| Z940 | Kidney transplant status |

**Table S2. Codes ICD 10 included in the diagnosis of Chronic liver disease.**

| **ICD 10** | **Label** |
| --- | --- |
| B18 | Chronic viral hepatitis |
| K70 | Alcoholic liver disease |
| K713 | Toxic liver disease with chronic persistent hepatitis |
| K714 | Toxic liver disease with chronic lobular hepatitis |
| K715 | Toxic liver disease with chronic active hepatitis |
| K717 | Toxic liver disease with fibrosis and cirrhosis of liver |
| K721 | Chronic hepatic failure |
| K73 | Chronic hepatitis, not elsewhere classified |
| K74 | Fibrosis and cirrhosis of liver |
| K753 | Granulomatous hepatitis, not elsewhere classified |
| K754 | Autoimmune hepatitis |
| K758 | Other specified inflammatory liver diseases |
| K761 | Chronic passive congestion of liver |
| K766 | Portal hypertension |
| K767 | Hepatorenal syndrome |
| K778 | Liver disorders in other diseases classified elsewhere |
| Q446 | Cystic disease of liver |
| Z944 | Liver transplant status |

**Table S3. List of duplicate therapy.**

| **Therapeutic group/Drug A** | **Therapeutic group/Drug B** |
| --- | --- |
| H2-receptor antagonists | H2-receptor antagonists |
| H2-receptor antagonists | Prostaglandins |
| H2-receptor antagonists | Proton pump inhibitors |
| H2-receptor antagonists | Naproxen and esomeprazole |
| Prostaglandins | Prostaglandins |
| Prostaglandins | Proton pump inhibitors |
| Prostaglandins | Naproxen and esomeprazole |
| Proton pump inhibitors | Proton pump inhibitors |
| Proton pump inhibitors | Naproxen and esomeprazole |
| Metformin | Metformin |
| Glibenclamide | Glibenclamide |
| Glibenclamide | Chlorpropamide |
| Glibenclamide | Glipizide |
| Glibenclamide | Gliquidone |
| Glibenclamide | Gliclazide |
| Glibenclamide | Glimepiride |
| Glibenclamide | Glisentide |
| Chlorpropamide | Chlorpropamide |
| Chlorpropamide | Glipizide |
| Chlorpropamide | Gliquidone |
| Chlorpropamide | Gliclazide |
| Chlorpropamide | Glimepiride |
| Chlorpropamide | Glisentide |
| Glipizide | Glipizide |
| Glipizide | Gliquidone |
| Glipizide | Gliclazide |
| Glipizide | Glimepiride |
| Glipizide | Glisentide |
| Gliquidone | Gliquidone |
| Gliquidone | Gliclazide |
| Gliquidone | Glimepiride |
| Gliquidone | Glisentide |
| Gliclazide | Glimepiride |
| Gliclazide | Glisentide |
| Glimepiride | Glisentide |
| Glisentide | Glisentide |
| Sulfonylureas | Glimepiride and pioglitazone |
| Metformin and pioglitazone | Metformin and pioglitazone |
| Metformin and pioglitazone | Metformin and sitagliptin |
| Metformin and pioglitazone | Metformin and vildagliptin |
| Metformin and pioglitazone | Metformin and saxagliptin |
| Metformin and pioglitazone | Metformin and linagliptin |
| Glimepiride and pioglitazone | Metformin and pioglitazone |
| Glimepiride and pioglitazone | Glimepiride and pioglitazone |
| Metformin and sitagliptin | Metformin and sitagliptin |
| Metformin and sitagliptin | Metformin and vildagliptin |
| Metformin and sitagliptin | Metformin and saxagliptin |
| Metformin and sitagliptin | Metformin and linagliptin |
| Metformin and sitagliptin | Metformin and alogliptin |
| Metformin and vildagliptin | Metformin and vildagliptin |
| Metformin and vildagliptin | Metformin and saxagliptin |
| Metformin and vildagliptin | Metformin and linagliptin |
| Metformin and vildagliptin | Metformin and alogliptin |
| Metformin and saxagliptin | Metformin and saxagliptin |
| Metformin and saxagliptin | Metformin and linagliptin |
| Metformin and saxagliptin | Metformin and alogliptin |
| Metformin and linagliptin | Metformin and linagliptin |
| Metformin and linagliptin | Metformin and alogliptin |
| Metformin and alogliptin | Metformin and alogliptin |
| Metformin and dapagliflozin | Metformin and dapagliflozin |
| Metformin and dapagliflozin | Metformin and canagliflozin |
| Metformin and dapagliflozin | Metformin and empagliflozin |
| Metformin and canagliflozin | Metformin and canagliflozin |
| Metformin and canagliflozin | Metformin and empagliflozin |
| Metformin and empagliflozin | Metformin and empagliflozin |
| Thiazolidinediones | Metformin and pioglitazone |
| Thiazolidinediones | Glimepiride and pioglitazone |
| Dipeptidyl peptidase 4 inhibitors | Metformin and sitagliptin |
| Dipeptidyl peptidase 4 inhibitors | Metformin and vildagliptin |
| Dipeptidyl peptidase 4 inhibitors | Metformin and saxagliptin |
| Dipeptidyl peptidase 4 inhibitors | Metformin and linagliptin |
| Dipeptidyl peptidase 4 inhibitors | Metformin and alogliptin |
| Dipeptidyl peptidase 4 inhibitors | Dipeptidyl peptidase 4 inhibitors |
| Glucagon-like peptide-1 analogues | Glucagon-like peptide-1 analogues |
| Sodium-glucose co-transporter 2 inhibitors | Metformin and dapagliflozin |
| Sodium-glucose co-transporter 2 inhibitors | Metformin and canagliflozin |
| Sodium-glucose co-transporter 2 inhibitors | Metformin and empagliflozin |
| Sodium-glucose co-transporter 2 inhibitors | Sodium-glucose co-transporter 2 inhibitors |
| Repaglinide | Nateglinide |
| Nateglinide | Nateglinide |
| Exenatide | Exenatide |
| Exenatide | Liraglutide |
| Exenatide | Lixisenatide |
| Exenatide | Albiglutide |
| Exenatide | Dulaglutide |
| Liraglutide | Liraglutide |
| Liraglutide | Lixisenatide |
| Liraglutide | Albiglutide |
| Liraglutide | Dulaglutide |
| Dapagliflozin | Metformin and dapagliflozin |
| Dapagliflozin | Metformin and canagliflozin |
| Dapagliflozin | Metformin and empagliflozin |
| Dapagliflozin | Dapagliflozin |
| Dapagliflozin | Canagliflozin |
| Dapagliflozin | Empagliflozin |
| Lixisenatide | Lixisenatide |
| Lixisenatide | Albiglutide |
| Lixisenatide | Dulaglutide |
| Canagliflozin | Metformin and canagliflozin |
| Canagliflozin | Metformin and empagliflozin |
| Canagliflozin | Canagliflozin |
| Canagliflozin | Empagliflozin |
| Empagliflozin | Metformin and empagliflozin |
| Empagliflozin | Empagliflozin |
| Albiglutide | Albiglutide |
| Albiglutide | Dulaglutide |
| Dulaglutide | Dulaglutide |
| Calcium | Calcium |
| Calcium | Calcium, combinations with vitamin D and/or other drugs |
| Calcium, combinations with vitamin D and/or other drugs | Calcium, combinations with vitamin D and/or other drugs |
| Potassium | Potassium |
| Warfarin | Acenocumarol |
| Vitamin K antagonists | Dabigatran etexilate |
| Vitamin K antagonists | Direct factor Xa inhibitors |
| Acetylsalicylic acid | Acetylsalicylic acid |
| Acetylsalicylic acid | Triflusal |
| Acetylsalicylic acid | Platelet aggregation inhibitors excl. Heparin, combinations |
| Acetylsalicylic acid | Atorvastatin, acetylsalicylic acid and ramipril |
| Acetylsalicylic acid | Acetylsalicylic acid |
| Acetylsalicylic acid | Acetylsalicylic acid, combinations excl. Psycholeptics |
| Triflusal | Triflusal |
| Triflusal | Platelet aggregation inhibitors excl. Heparin, combinations |
| Platelet aggregation inhibitors excl. Heparin, combinations | Platelet aggregation inhibitors excl. Heparin, combinations |
| Platelet aggregation inhibitors excl. Heparin, combinations | Atorvastatin, acetylsalicylic acid and ramipril |
| Dabigatran etexilate | Dabigatran etexilate |
| Dabigatran etexilate | Direct factor Xa inhibitors |
| Direct factor Xa inhibitors | Direct factor Xa inhibitors |
| Iron bivalent, oral preparations | Iron bivalent, oral preparations |
| Iron bivalent, oral preparations | Iron trivalent, oral preparations |
| Iron bivalent, oral preparations | Iron in combination with folic acid |
| Iron bivalent, oral preparations | Iron in other combinations |
| Iron trivalent, oral preparations | Iron trivalent, oral preparations |
| Iron trivalent, oral preparations | Iron in combination with folic acid |
| Iron trivalent, oral preparations | Iron in other combinations |
| Iron in combination with folic acid | Iron in combination with folic acid |
| Iron in combination with folic acid | Iron in other combinations |
| Iron in other combinations | Iron in other combinations |
| Digitalis glycosides | Digitalis glycosides |
| Antiarrhythmics, class Ia | Antiarrhythmics, class Ia |
| Antiarrhythmics, class Ia | Antiarrhythmics, class Ic |
| Antiarrhythmics, class Ia | Antiarrhythmics, class III |
| Flecainide | Flecainide |
| Antiarrhythmics, class Ic | Antiarrhythmics, class III |
| Antiarrhythmics, class III | Antiarrhythmics, class III |
| Prazosin | Doxazosin |
| Prazosin | Terazosin |
| Doxazosin | Terazosin |
| Terazosin | Terazosin |
| Alpha-adrenoreceptor antagonists | Alpha-adrenoreceptor antagonists |
| Thiazides, plain | Thiazides, plain |
| Thiazides, plain | Thiazides, combinations with other drugs |
| Thiazides, plain | Beta blocking agents, selective, and thiazides |
| Thiazides, plain | ACE inhibitors and diuretics |
| Thiazides, plain | Angiotensin II antagonists and diuretics |
| Thiazides, plain | Valsartan, amlodipine and hydrochlorothiazide |
| Thiazides, plain | Olmesartan medoxomil, amlodipine and hydrochlorothiazide |
| Thiazides, combinations with other drugs | Thiazides, combinations with other drugs |
| Thiazides, combinations with other drugs | Beta blocking agents, selective, and thiazides |
| Thiazides, combinations with other drugs | ACE inhibitors and diuretics |
| Thiazides, combinations with other drugs | Angiotensin II antagonists and diuretics |
| Thiazides, combinations with other drugs | Valsartan, amlodipine and hydrochlorothiazide |
| Thiazides, combinations with other drugs | Olmesartan medoxomil, amlodipine and hydrochlorothiazide |
| Sulfonamides, plain | Sulfonamides, plain |
| Chlortalidone | Bumetanide |
| Chlortalidone | Piretanide |
| Chlortalidone | Torasemide |
| Xipamide | Furosemide |
| Xipamide | Bumetanide |
| Xipamide | Piretanide |
| Xipamide | Torasemide |
| Xipamide | Bumetanide |
| Indapamide | Furosemide |
| Indapamide | Bumetanide |
| Indapamide | Piretanide |
| Indapamide | Torasemide |
| Sulfonamides, plain | Chlortalidone and potassium-sparing agents |
| Sulfonamides, plain | High-ceiling diuretics and potassium-sparing agents |
| Furosemide | Furosemide |
| Furosemide | Bumetanide |
| Furosemide | Piretanide |
| Furosemide | Torasemide |
| Bumetanide | Bumetanide |
| Bumetanide | Piretanide |
| Bumetanide | Torasemide |
| Piretanide | Piretanide |
| Piretanide | Torasemide |
| Sulfonamides, plain | Chlortalidone and potassium-sparing agents |
| Sulfonamides, plain | High-ceiling diuretics and potassium-sparing agents |
| Aldosterone antagonists | Aldosterone antagonists |
| Aldosterone antagonists | Low-ceiling diuretics and potassium-sparing agents |
| Aldosterone antagonists | High-ceiling diuretics and potassium-sparing agents |
| Low-ceiling diuretics and potassium-sparing agents | Low-ceiling diuretics and potassium-sparing agents |
| Low-ceiling diuretics and potassium-sparing agents | High-ceiling diuretics and potassium-sparing agents |
| High-ceiling diuretics and potassium-sparing agents | High-ceiling diuretics and potassium-sparing agents |
| High-ceiling diuretics and potassium-sparing agents | High-ceiling diuretics and potassium-sparing agents |
| Oxprenolol | Oxprenolol |
| Oxprenolol | Propranolol |
| Oxprenolol | Sotalol |
| Oxprenolol | Nadolol |
| Propranolol | Sotalol |
| Propranolol | Nadolol |
| Sotalol | Sotalol |
| Sotalol | Nadolol |
| Beta blocking agents, non-selective | Beta blocking agents, selective |
| Beta blocking agents, non-selective | Alpha and beta blocking agents |
| Beta blocking agents, non-selective | Beta blocking agents, selective, and thiazides |
| Beta blocking agents, non-selective | Beta blocking agents, selective, and other diuretics |
| Beta blocking agents, non-selective | Beta blocking agents, selective, thiazides and other diuretics |
| Beta blocking agents, non-selective | Beta blocking agents and calcium channel blockers |
| Metoprolol | Metoprolol |
| Metoprolol | Atenolol |
| Metoprolol | Bisoprolol |
| Metoprolol | Celiprolol |
| Metoprolol | Nebivolol |
| Atenolol | Atenolol |
| Atenolol | Bisoprolol |
| Atenolol | Celiprolol |
| Atenolol | Nebivolol |
| Bisoprolol | Celiprolol |
| Bisoprolol | Nebivolol |
| Celiprolol | Celiprolol |
| Celiprolol | Celiprolol |
| Celiprolol | Celiprolol |
| Beta blocking agents, selective | Alpha and beta blocking agents |
| Metoprolol | Atenolol and thiazides |
| Metoprolol | Bisoprolol and thiazides |
| Metoprolol | Nebivolol and thiazides |
| Atenolol | Atenolol and thiazides |
| Atenolol | Bisoprolol and thiazides |
| Atenolol | Nebivolol and thiazides |
| Bisoprolol | Atenolol and thiazides |
| Bisoprolol | Nebivolol and thiazides |
| Celiprolol | Atenolol and thiazides |
| Celiprolol | Bisoprolol and thiazides |
| Celiprolol | Nebivolol and thiazides |
| Nebivolol | Atenolol and thiazides |
| Nebivolol | Bisoprolol and thiazides |
| Nebivolol | Nebivolol and thiazides |
| Beta blocking agents, selective | Beta blocking agents, selective, and other diuretics |
| Beta blocking agents, selective | Beta blocking agents, selective, thiazides and other diuretics |
| Beta blocking agents, selective | Beta blocking agents and calcium channel blockers |
| Labetalol | Carvedilol |
| Alpha and beta blocking agents | Beta blocking agents, selective, and thiazides |
| Alpha and beta blocking agents | Beta blocking agents, selective, and other diuretics |
| Alpha and beta blocking agents | Beta blocking agents, selective, thiazides and other diuretics |
| Alpha and beta blocking agents | Beta blocking agents and calcium channel blockers |
| Beta blocking agents, selective, and thiazides | Beta blocking agents, selective, and thiazides |
| Beta blocking agents, selective, and thiazides | Beta blocking agents, selective, and thiazides |
| Beta blocking agents, selective, and thiazides | Beta blocking agents, selective, and other diuretics |
| Beta blocking agents, selective, and thiazides | Beta blocking agents, selective, thiazides and other diuretics |
| Beta blocking agents, selective, and thiazides | Beta blocking agents and calcium channel blockers |
| Atenolol and thiazides | ACE inhibitors and diuretics |
| Atenolol and thiazides | Angiotensin II antagonists and diuretics |
| Atenolol and thiazides | Valsartan, amlodipine and hydrochlorothiazide |
| Atenolol and thiazides | Olmesartan medoxomil, amlodipine and hydrochlorothiazide |
| Beta blocking agents, selective, and other diuretics | Beta blocking agents, selective, and other diuretics |
| Beta blocking agents, selective, and other diuretics | Beta blocking agents, selective, thiazides and other diuretics |
| Beta blocking agents, selective, and other diuretics | Beta blocking agents and calcium channel blockers |
| Beta blocking agents, selective, thiazides and other diuretics | Beta blocking agents, selective, thiazides and other diuretics |
| Beta blocking agents, selective, thiazides and other diuretics | Beta blocking agents and calcium channel blockers |
| Beta blocking agents and calcium channel blockers | Beta blocking agents and calcium channel blockers |
| Amlodipine | Amlodipine |
| Amlodipine | Felodipine |
| Amlodipine | Isradipine |
| Amlodipine | Nicardipine |
| Amlodipine | Nifedipine |
| Amlodipine | Nimodipine |
| Amlodipine | Nisoldipine |
| Amlodipine | Nitrendipine |
| Amlodipine | Lacidipine |
| Amlodipine | Manidipine |
| Amlodipine | Barnidipine |
| Amlodipine | Lercanidipine |
| Felodipine | Felodipine |
| Felodipine | Isradipine |
| Felodipine | Nicardipine |
| Felodipine | Nifedipine |
| Felodipine | Nimodipine |
| Felodipine | Nisoldipine |
| Felodipine | Nitrendipine |
| Felodipine | Lacidipine |
| Felodipine | Manidipine |
| Felodipine | Barnidipine |
| Felodipine | Lercanidipine |
| Isradipine | Isradipine |
| Isradipine | Nicardipine |
| Isradipine | Nifedipine |
| Isradipine | Nimodipine |
| Isradipine | Nisoldipine |
| Isradipine | Nitrendipine |
| Isradipine | Lacidipine |
| Isradipine | Manidipine |
| Isradipine | Barnidipine |
| Isradipine | Lercanidipine |
| Nicardipine | Nicardipine |
| Nicardipine | Nifedipine |
| Nicardipine | Nimodipine |
| Nicardipine | Nisoldipine |
| Nicardipine | Nitrendipine |
| Nicardipine | Lacidipine |
| Nicardipine | Manidipine |
| Nicardipine | Barnidipine |
| Nicardipine | Lercanidipine |
| Nifedipine | Nimodipine |
| Nifedipine | Nisoldipine |
| Nifedipine | Nitrendipine |
| Nifedipine | Lacidipine |
| Nifedipine | Manidipine |
| Nifedipine | Barnidipine |
| Nifedipine | Lercanidipine |
| Nimodipine | Nimodipine |
| Nimodipine | Nisoldipine |
| Nimodipine | Nitrendipine |
| Nimodipine | Lacidipine |
| Nimodipine | Manidipine |
| Nimodipine | Barnidipine |
| Nimodipine | Lercanidipine |
| Nisoldipine | Nisoldipine |
| Nisoldipine | Nitrendipine |
| Nisoldipine | Lacidipine |
| Nisoldipine | Manidipine |
| Nisoldipine | Barnidipine |
| Nisoldipine | Lercanidipine |
| Nitrendipine | Nitrendipine |
| Nitrendipine | Lacidipine |
| Nitrendipine | Manidipine |
| Nitrendipine | Barnidipine |
| Nitrendipine | Lercanidipine |
| Lacidipine | Lacidipine |
| Lacidipine | Manidipine |
| Lacidipine | Barnidipine |
| Lacidipine | Lercanidipine |
| Manidipine | Manidipine |
| Manidipine | Barnidipine |
| Manidipine | Lercanidipine |
| Barnidipine | Barnidipine |
| Barnidipine | Lercanidipine |
| Lercanidipine | Lercanidipine |
| Dihydropyridine derivatives | ACE inhibitors and calcium channel blockers |
| Dihydropyridine derivatives | Angiotensin II antagonists and calcium channel blockers |
| Dihydropyridine derivatives | Valsartan, amlodipine and hydrochlorothiazide |
| Dihydropyridine derivatives | Olmesartan medoxomil, amlodipine and hydrochlorothiazide |
| Dihydropyridine derivatives | Atorvastatin and amlodipine |
| Phenylalkylamine derivatives | Benzothiazepine derivatives |
| Phenylalkylamine derivatives | Trandolapril and verapamil |
| Benzothiazepine derivatives | Trandolapril and verapamil |
| Captopril | Enalapril |
| Captopril | Lisinopril |
| Captopril | Perindopril |
| Captopril | Ramipril |
| Captopril | Quinapril |
| Captopril | Benazepril |
| Captopril | Cilazapril |
| Captopril | Fosinopril |
| Captopril | Trandolapril |
| Captopril | Spirapril |
| Captopril | Delapril |
| Captopril | Zofenopril |
| Captopril | Imidapril |
| Enalapril | Lisinopril |
| Enalapril | Perindopril |
| Enalapril | Ramipril |
| Enalapril | Quinapril |
| Enalapril | Benazepril |
| Enalapril | Cilazapril |
| Enalapril | Fosinopril |
| Enalapril | Trandolapril |
| Enalapril | Spirapril |
| Enalapril | Delapril |
| Enalapril | Zofenopril |
| Enalapril | Imidapril |
| Lisinopril | Perindopril |
| Lisinopril | Ramipril |
| Lisinopril | Quinapril |
| Lisinopril | Benazepril |
| Lisinopril | Cilazapril |
| Lisinopril | Fosinopril |
| Lisinopril | Trandolapril |
| Lisinopril | Spirapril |
| Lisinopril | Delapril |
| Lisinopril | Zofenopril |
| Lisinopril | Imidapril |
| Perindopril | Ramipril |
| Perindopril | Quinapril |
| Perindopril | Benazepril |
| Perindopril | Cilazapril |
| Perindopril | Fosinopril |
| Perindopril | Trandolapril |
| Perindopril | Spirapril |
| Perindopril | Delapril |
| Perindopril | Zofenopril |
| Perindopril | Imidapril |
| Ramipril | Quinapril |
| Ramipril | Benazepril |
| Ramipril | Cilazapril |
| Ramipril | Fosinopril |
| Ramipril | Trandolapril |
| Ramipril | Spirapril |
| Ramipril | Delapril |
| Ramipril | Zofenopril |
| Ramipril | Imidapril |
| Quinapril | Benazepril |
| Quinapril | Cilazapril |
| Quinapril | Fosinopril |
| Quinapril | Trandolapril |
| Quinapril | Spirapril |
| Quinapril | Delapril |
| Quinapril | Zofenopril |
| Quinapril | Imidapril |
| Benazepril | Cilazapril |
| Benazepril | Fosinopril |
| Benazepril | Trandolapril |
| Benazepril | Spirapril |
| Benazepril | Delapril |
| Benazepril | Zofenopril |
| Benazepril | Imidapril |
| Cilazapril | Fosinopril |
| Cilazapril | Trandolapril |
| Cilazapril | Spirapril |
| Cilazapril | Delapril |
| Cilazapril | Zofenopril |
| Cilazapril | Imidapril |
| Fosinopril | Trandolapril |
| Fosinopril | Spirapril |
| Fosinopril | Delapril |
| Fosinopril | Zofenopril |
| Fosinopril | Imidapril |
| Trandolapril | Spirapril |
| Trandolapril | Delapril |
| Trandolapril | Zofenopril |
| Trandolapril | Imidapril |
| Spirapril | Delapril |
| Spirapril | Zofenopril |
| Spirapril | Imidapril |
| Delapril | Zofenopril |
| Delapril | Imidapril |
| Zofenopril | Imidapril |
| Captopril | Enalapril and diuretics |
| Captopril | Lisinopril and diuretics |
| Captopril | Perindopril and diuretics |
| Captopril | Ramipril and diuretics |
| Captopril | Quinapril and diuretics |
| Captopril | Benazepril and diuretics |
| Captopril | Cilazapril and diuretics |
| Captopril | Fosinopril and diuretics |
| Captopril | Zofenopril and diuretics |
| Enalapril | Captopril and diuretics |
| Enalapril | Lisinopril and diuretics |
| Enalapril | Perindopril and diuretics |
| Enalapril | Ramipril and diuretics |
| Enalapril | Quinapril and diuretics |
| Enalapril | Benazepril and diuretics |
| Enalapril | Cilazapril and diuretics |
| Enalapril | Fosinopril and diuretics |
| Enalapril | Zofenopril and diuretics |
| Lisinopril | Captopril and diuretics |
| Lisinopril | Enalapril and diuretics |
| Lisinopril | Perindopril and diuretics |
| Lisinopril | Ramipril and diuretics |
| Lisinopril | Quinapril and diuretics |
| Lisinopril | Benazepril and diuretics |
| Lisinopril | Cilazapril and diuretics |
| Lisinopril | Fosinopril and diuretics |
| Lisinopril | Zofenopril and diuretics |
| Perindopril | Captopril and diuretics |
| Perindopril | Enalapril and diuretics |
| Perindopril | Lisinopril and diuretics |
| Perindopril | Ramipril and diuretics |
| Perindopril | Quinapril and diuretics |
| Perindopril | Benazepril and diuretics |
| Perindopril | Cilazapril and diuretics |
| Perindopril | Fosinopril and diuretics |
| Perindopril | Zofenopril and diuretics |
| Ramipril | Captopril and diuretics |
| Ramipril | Enalapril and diuretics |
| Ramipril | Lisinopril and diuretics |
| Ramipril | Perindopril and diuretics |
| Ramipril | Quinapril and diuretics |
| Ramipril | Benazepril and diuretics |
| Ramipril | Cilazapril and diuretics |
| Ramipril | Fosinopril and diuretics |
| Ramipril | Zofenopril and diuretics |
| Quinapril | Captopril and diuretics |
| Quinapril | Enalapril and diuretics |
| Quinapril | Lisinopril and diuretics |
| Quinapril | Perindopril and diuretics |
| Quinapril | Ramipril and diuretics |
| Quinapril | Benazepril and diuretics |
| Quinapril | Cilazapril and diuretics |
| Quinapril | Fosinopril and diuretics |
| Quinapril | Zofenopril and diuretics |
| Benazepril | Captopril and diuretics |
| Benazepril | Enalapril and diuretics |
| Benazepril | Lisinopril and diuretics |
| Benazepril | Perindopril and diuretics |
| Benazepril | Ramipril and diuretics |
| Benazepril | Quinapril and diuretics |
| Benazepril | Cilazapril and diuretics |
| Benazepril | Fosinopril and diuretics |
| Benazepril | Zofenopril and diuretics |
| Benazepril | Captopril and diuretics |
| Benazepril | Enalapril and diuretics |
| Cilazapril | Lisinopril and diuretics |
| Cilazapril | Perindopril and diuretics |
| Cilazapril | Ramipril and diuretics |
| Cilazapril | Quinapril and diuretics |
| Cilazapril | Benazepril and diuretics |
| Cilazapril | Fosinopril and diuretics |
| Cilazapril | Zofenopril and diuretics |
| Fosinopril | Captopril and diuretics |
| Fosinopril | Enalapril and diuretics |
| Fosinopril | Lisinopril and diuretics |
| Fosinopril | Perindopril and diuretics |
| Fosinopril | Ramipril and diuretics |
| Fosinopril | Quinapril and diuretics |
| Fosinopril | Benazepril and diuretics |
| Fosinopril | Cilazapril and diuretics |
| Fosinopril | Zofenopril and diuretics |
| Trandolapril | ACE inhibitors, combinations |
| Spirapril | ACE inhibitors, combinations |
| Delapril | ACE inhibitors, combinations |
| Zofenopril | Captopril and diuretics |
| Zofenopril | Enalapril and diuretics |
| Zofenopril | Lisinopril and diuretics |
| Zofenopril | Perindopril and diuretics |
| Zofenopril | Ramipril and diuretics |
| Zofenopril | Quinapril and diuretics |
| Zofenopril | Benazepril and diuretics |
| Zofenopril | Cilazapril and diuretics |
| Zofenopril | Fosinopril and diuretics |
| Imidapril | ACE inhibitors, combinations |
| Captopril | ACE inhibitors and calcium channel blockers |
| Enalapril | Ramipril and felodipine |
| Enalapril | Enalapril and nitrendipine |
| Enalapril | Trandolapril and verapamil |
| Enalapril | Delapril and manidipine |
| Lisinopril | ACE inhibitors and calcium channel blockers |
| Perindopril | ACE inhibitors and calcium channel blockers |
| Ramipril | Enalapril and lercanidipine |
| Ramipril | Enalapril and nitrendipine |
| Ramipril | Trandolapril and verapamil |
| Ramipril | Delapril and manidipine |
| Quinapril | ACE inhibitors and calcium channel blockers |
| Benazepril | ACE inhibitors and calcium channel blockers |
| Cilazapril | ACE inhibitors and calcium channel blockers |
| Fosinopril | ACE inhibitors and calcium channel blockers |
| Trandolapril | Enalapril and lercanidipine |
| Trandolapril | Ramipril and felodipine |
| Trandolapril | Enalapril and nitrendipine |
| Trandolapril | Delapril and manidipine |
| Spirapril | ACE inhibitors and calcium channel blockers |
| Delapril | Enalapril and lercanidipine |
| Delapril | Ramipril and felodipine |
| Delapril | Enalapril and nitrendipine |
| Delapril | Trandolapril and verapamil |
| Zofenopril | ACE inhibitors and calcium channel blockers |
| Imidapril | ACE inhibitors and calcium channel blockers |
| ACE inhibitors and diuretics | ACE inhibitors and diuretics |
| ACE inhibitors and diuretics | ACE inhibitors and diuretics |
| ACE inhibitors and diuretics | ACE inhibitors and calcium channel blockers |
| ACE inhibitors and diuretics | Angiotensin II antagonists and diuretics |
| ACE inhibitors and calcium channel blockers | ACE inhibitors and calcium channel blockers |
| ACE inhibitors and calcium channel blockers | Angiotensin II antagonists and calcium channel blockers |
| ACE inhibitors and calcium channel blockers | Valsartan, amlodipine and hydrochlorothiazide |
| ACE inhibitors and calcium channel blockers | Olmesartan medoxomil, amlodipine and hydrochlorothiazide |
| ACE inhibitors and calcium channel blockers | Atorvastatin and amlodipine |
| Losartan | Eprosartan |
| Losartan | Valsartan |
| Losartan | Irbesartan |
| Losartan | Candesartan |
| Losartan | Telmisartan |
| Losartan | Olmesartan medoxomil |
| Eprosartan | Valsartan |
| Eprosartan | Irbesartan |
| Eprosartan | Candesartan |
| Eprosartan | Telmisartan |
| Eprosartan | Olmesartan medoxomil |
| Valsartan | Irbesartan |
| Valsartan | Candesartan |
| Valsartan | Telmisartan |
| Valsartan | Olmesartan medoxomil |
| Irbesartan | Candesartan |
| Irbesartan | Telmisartan |
| Irbesartan | Olmesartan medoxomil |
| Candesartan | Telmisartan |
| Candesartan | Olmesartan medoxomil |
| Telmisartan | Olmesartan medoxomil |
| Losartan | Eprosartan and diuretics |
| Losartan | Valsartan and diuretics |
| Losartan | Irbesartan and diuretics |
| Losartan | Candesartan and diuretics |
| Losartan | Telmisartan and diuretics |
| Losartan | Olmesartan medoxomil and diuretics |
| Eprosartan | Losartan and diuretics |
| Eprosartan | Valsartan and diuretics |
| Eprosartan | Irbesartan and diuretics |
| Eprosartan | Candesartan and diuretics |
| Eprosartan | Telmisartan and diuretics |
| Eprosartan | Olmesartan medoxomil and diuretics |
| Valsartan | Losartan and diuretics |
| Valsartan | Eprosartan and diuretics |
| Valsartan | Irbesartan and diuretics |
| Valsartan | Candesartan and diuretics |
| Valsartan | Telmisartan and diuretics |
| Valsartan | Olmesartan medoxomil and diuretics |
| Irbesartan | Losartan and diuretics |
| Irbesartan | Eprosartan and diuretics |
| Irbesartan | Valsartan and diuretics |
| Irbesartan | Candesartan and diuretics |
| Irbesartan | Telmisartan and diuretics |
| Irbesartan | Olmesartan medoxomil and diuretics |
| Candesartan | Losartan and diuretics |
| Candesartan | Eprosartan and diuretics |
| Candesartan | Valsartan and diuretics |
| Candesartan | Irbesartan and diuretics |
| Candesartan | Telmisartan and diuretics |
| Candesartan | Olmesartan medoxomil and diuretics |
| Telmisartan | Losartan and diuretics |
| Telmisartan | Eprosartan and diuretics |
| Telmisartan | Valsartan and diuretics |
| Telmisartan | Irbesartan and diuretics |
| Telmisartan | Candesartan and diuretics |
| Telmisartan | Olmesartan medoxomil and diuretics |
| Olmesartan medoxomil | Losartan and diuretics |
| Olmesartan medoxomil | Eprosartan and diuretics |
| Olmesartan medoxomil | Valsartan and diuretics |
| Olmesartan medoxomil | Irbesartan and diuretics |
| Olmesartan medoxomil | Candesartan and diuretics |
| Olmesartan medoxomil | Telmisartan and diuretics |
| Angiotensin II antagonists, plain | Angiotensin II antagonists and calcium channel blockers |
| Losartan | Angiotensin II antagonists and calcium channel blockers |
| Eprosartan | Angiotensin II antagonists and calcium channel blockers |
| Valsartan | Olmesartan medoxomil and amlodipine |
| Valsartan | Telmisartan and amlodipine |
| Irbesartan | Angiotensin II antagonists and calcium channel blockers |
| Candesartan | Angiotensin II antagonists and calcium channel blockers |
| Telmisartan | Valsartan and amlodipine |
| Telmisartan | Olmesartan medoxomil and amlodipine |
| Olmesartan medoxomil | Valsartan and amlodipine |
| Olmesartan medoxomil | Telmisartan and amlodipine |
| Losartan | Angiotensin II antagonists, other combinations |
| Eprosartan | Angiotensin II antagonists, other combinations |
| Valsartan | Olmesartan medoxomil, amlodipine and hydrochlorothiazide |
| Irbesartan | Angiotensin II antagonists, other combinations |
| Candesartan | Angiotensin II antagonists, other combinations |
| Telmisartan | Angiotensin II antagonists, other combinations |
| Olmesartan medoxomil | Valsartan, amlodipine and hydrochlorothiazide |
| Angiotensin II antagonists and diuretics | Angiotensin II antagonists and diuretics |
| Angiotensin II antagonists and diuretics | Angiotensin II antagonists and diuretics |
| Angiotensin II antagonists and diuretics | Angiotensin II antagonists and calcium channel blockers |
| Angiotensin II antagonists and diuretics | Angiotensin II antagonists, other combinations |
| Angiotensin II antagonists and calcium channel blockers | Angiotensin II antagonists and calcium channel blockers |
| Angiotensin II antagonists and calcium channel blockers | Valsartan, amlodipine and hydrochlorothiazide |
| Angiotensin II antagonists and calcium channel blockers | Olmesartan medoxomil, amlodipine and hydrochlorothiazide |
| Angiotensin II antagonists and calcium channel blockers | Atorvastatin and amlodipine |
| Angiotensin II antagonists, other combinations | Angiotensin II antagonists, other combinations |
| Valsartan, amlodipine and hydrochlorothiazide | Atorvastatin and amlodipine |
| Olmesartan medoxomil, amlodipine and hydrochlorothiazide | Atorvastatin and amlodipine |
| HMG CoA reductase inhibitors | HMG CoA reductase inhibitors |
| HMG CoA reductase inhibitors | HMG CoA reductase inhibitors in combination with other lipid modifying agents |
| HMG CoA reductase inhibitors | HMG CoA reductase inhibitors, other combinations |
| Bezafibrate | Bezafibrate |
| Bezafibrate | Gemfibrozil |
| Bezafibrate | Fenofibrate |
| Gemfibrozil | Fenofibrate |
| Fenofibrate | Fenofibrate |
| Fibrates | Pravastatin and fenofibrate |
| Ezetimibe | Ezetimibe |
| Ezetimibe | Simvastatin and ezetimibe |
| Ezetimibe | Atorvastatin and ezetimibe |
| HMG CoA reductase inhibitors in combination with other lipid modifying agents | HMG CoA reductase inhibitors in combination with other lipid modifying agents |
| HMG CoA reductase inhibitors, other combinations | HMG CoA reductase inhibitors in combination with other lipid modifying agents |
| HMG CoA reductase inhibitors, other combinations | HMG CoA reductase inhibitors, other combinations |
| Selective estrogen receptor modulators | Selective estrogen receptor modulators |
| Drugs for urinary frequency and incontinence | Drugs for urinary frequency and incontinence |
| Drugs for urinary frequency and incontinence | Tamsulosin and dutasteride |
| Alfuzosin | Alfuzosin |
| Alfuzosin | Tamsulosin |
| Alfuzosin | Terazosin |
| Alfuzosin | Silodosin |
| Alfuzosin | Tamsulosin and dutasteride |
| Tamsulosin | Tamsulosin |
| Tamsulosin | Terazosin |
| Tamsulosin | Silodosin |
| Tamsulosin | Tamsulosin and dutasteride |
| Terazosin | Silodosin |
| Terazosin | Tamsulosin and dutasteride |
| Silodosin | Silodosin |
| Silodosin | Tamsulosin and dutasteride |
| Tamsulosin and dutasteride | Tamsulosin and dutasteride |
| Testosterone-5-alpha reductase inhibitors | Tamsulosin and dutasteride |
| Testosterone-5-alpha reductase inhibitors | Testosterone-5-alpha reductase inhibitors |
| Mineralocorticoids | Mineralocorticoids |
| Mineralocorticoids | Betamethasone |
| Mineralocorticoids | Prednisolone |
| Mineralocorticoids | Triamcinolone |
| Mineralocorticoids | Corticosteroids for systemic use, combinations |
| Betamethasone | Betamethasone |
| Betamethasone | Dexamethasone |
| Betamethasone | Methylprednisolone |
| Betamethasone | Prednisolone |
| Betamethasone | Prednisone |
| Betamethasone | Triamcinolone |
| Betamethasone | Hydrocortisone |
| Betamethasone | Deflazacort |
| Dexamethasone | Methylprednisolone |
| Dexamethasone | Prednisolone |
| Dexamethasone | Prednisone |
| Dexamethasone | Triamcinolone |
| Dexamethasone | Hydrocortisone |
| Dexamethasone | Deflazacort |
| Methylprednisolone | Prednisolone |
| Methylprednisolone | Prednisone |
| Methylprednisolone | Triamcinolone |
| Methylprednisolone | Hydrocortisone |
| Methylprednisolone | Deflazacort |
| Prednisolone | Prednisolone |
| Prednisolone | Prednisone |
| Prednisolone | Triamcinolone |
| Prednisolone | Hydrocortisone |
| Prednisolone | Deflazacort |
| Prednisone | Triamcinolone |
| Prednisone | Hydrocortisone |
| Prednisone | Deflazacort |
| Triamcinolone | Triamcinolone |
| Triamcinolone | Hydrocortisone |
| Triamcinolone | Deflazacort |
| Hydrocortisone | Hydrocortisone |
| Hydrocortisone | Deflazacort |
| Glucocorticoids | Corticosteroids for systemic use, combinations |
| Corticosteroids for systemic use, combinations | Corticosteroids for systemic use, combinations |
| Parathyroid hormones and analogues | Selective estrogen receptor modulators |
| Parathyroid hormones and analogues | Parathyroid hormones and analogues |
| Parathyroid hormones and analogues | Calcitonin preparations |
| Parathyroid hormones and analogues | Bisphosphonates |
| Parathyroid hormones and analogues | Bisphosphonates, combinations |
| Parathyroid hormones and analogues | Other drugs affecting bone structure and mineralization |
| Calcitonin preparations | Selective estrogen receptor modulators |
| Calcitonin preparations | Calcitonin preparations |
| Calcitonin preparations | Bisphosphonates |
| Calcitonin preparations | Bisphosphonates, combinations |
| Calcitonin preparations | Other drugs affecting bone structure and mineralization |
| Tetracyclines | Tetracyclines |
| Ampicillin | Ampicillin |
| Ampicillin | Amoxicillin |
| Ampicillin | Ampicillin, combinations |
| Amoxicillin | Ampicillin, combinations |
| Ampicillin, combinations | Ampicillin, combinations |
| Penicillins with extended spectrum | Beta-lactamase sensitive penicillins |
| Penicillins with extended spectrum | Beta-lactamase resistant penicillins |
| Penicillins with extended spectrum | Combinations of penicillins, incl. beta-lactamase inhibitors |
| Beta-lactamase sensitive penicillins | Beta-lactamase sensitive penicillins |
| Beta-lactamase sensitive penicillins | Beta-lactamase resistant penicillins |
| Beta-lactamase sensitive penicillins | Combinations of penicillins, incl. Beta-lactamase inhibitors |
| Beta-lactamase resistant penicillins | Beta-lactamase resistant penicillins |
| Beta-lactamase resistant penicillins | Combinations of penicillins, incl. Beta-lactamase inhibitors |
| Combinations of penicillins, incl. Beta-lactamase inhibitors | Combinations of penicillins, incl. Beta-lactamase inhibitors |
| Beta-lactam antibacterials, penicillins | Other beta-lactam antibacterials |
| Other beta-lactam antibacterials | Other beta-lactam antibacterials |
| Macrolides | Macrolides |
| Quinolone antibacterials | Quinolone antibacterials |
| Aromatase inhibitors | Aromatase inhibitors |
| Butylpyrazolidines | Butylpyrazolidines |
| Butylpyrazolidines | Acetic acid derivatives and related substances |
| Butylpyrazolidines | Oxicams |
| Butylpyrazolidines | Propionic acid derivatives |
| Butylpyrazolidines | Fenamates |
| Butylpyrazolidines | Coxibs |
| Butylpyrazolidines | Nabumetone |
| Butylpyrazolidines | Niflumic acid |
| Butylpyrazolidines | Morniflumate |
| Butylpyrazolidines | Oxaceprol |
| Butylpyrazolidines | Isonixin |
| Butylpyrazolidines | Antiinflammatory/antirheumatic agents in combination with corticosteroids |
| Indometacin | Diclofenac |
| Indometacin | Aceclofenac |
| Indometacin | Diclofenac, combinations |
| Diclofenac | Diclofenac |
| Diclofenac | Aceclofenac |
| Diclofenac | Diclofenac, combinations |
| Aceclofenac | Aceclofenac |
| Aceclofenac | Diclofenac, combinations |
| Diclofenac, combinations | Diclofenac, combinations |
| Acetic acid derivatives and related substances | Oxicams |
| Acetic acid derivatives and related substances | Propionic acid derivatives |
| Acetic acid derivatives and related substances | Fenamates |
| Acetic acid derivatives and related substances | Coxibs |
| Acetic acid derivatives and related substances | Nabumetone |
| Acetic acid derivatives and related substances | Niflumic acid |
| Acetic acid derivatives and related substances | Morniflumate |
| Acetic acid derivatives and related substances | Oxaceprol |
| Acetic acid derivatives and related substances | Isonixin |
| Acetic acid derivatives and related substances | Antiinflammatory/antirheumatic agents in combination with corticosteroids |
| Oxicams | Oxicams |
| Oxicams | Propionic acid derivatives |
| Oxicams | Fenamates |
| Oxicams | Coxibs |
| Oxicams | Nabumetone |
| Oxicams | Niflumic acid |
| Oxicams | Morniflumate |
| Oxicams | Oxaceprol |
| Oxicams | Isonixin |
| Oxicams | Antiinflammatory/antirheumatic agents in combination with corticosteroids |
| Propionic acid derivatives | Propionic acid derivatives |
| Propionic acid derivatives | Fenamates |
| Propionic acid derivatives | Coxibs |
| Propionic acid derivatives | Nabumetone |
| Propionic acid derivatives | Niflumic acid |
| Propionic acid derivatives | Morniflumate |
| Propionic acid derivatives | Oxaceprol |
| Propionic acid derivatives | Isonixin |
| Propionic acid derivatives | Antiinflammatory/antirheumatic agents in combination with corticosteroids |
| Fenamates | Fenamates |
| Fenamates | Coxibs |
| Fenamates | Nabumetone |
| Fenamates | Niflumic acid |
| Fenamates | Morniflumate |
| Fenamates | Oxaceprol |
| Fenamates | Isonixin |
| Fenamates | Antiinflammatory/antirheumatic agents in combination with corticosteroids |
| Coxibs | Coxibs |
| Coxibs | Nabumetone |
| Coxibs | Niflumic acid |
| Coxibs | Morniflumate |
| Coxibs | Oxaceprol |
| Coxibs | Isonixin |
| Coxibs | Antiinflammatory/antirheumatic agents in combination with corticosteroids |
| Nabumetone | Nabumetone |
| Nabumetone | Niflumic acid |
| Nabumetone | Morniflumate |
| Nabumetone | Oxaceprol |
| Nabumetone | Isonixin |
| Niflumic acid | Niflumic acid |
| Niflumic acid | Morniflumate |
| Niflumic acid | Isonixin |
| Morniflumate | Morniflumate |
| Oxaceprol | Niflumic acid |
| Oxaceprol | Morniflumate |
| Oxaceprol | Oxaceprol |
| Oxaceprol | Isonixin |
| Isonixin | Morniflumate |
| Isonixin | Isonixin |
| Antiinflammatory/antirheumatic agents in combination with corticosteroids | Nabumetone |
| Antiinflammatory/antirheumatic agents in combination with corticosteroids | Niflumic acid |
| Antiinflammatory/antirheumatic agents in combination with corticosteroids | Morniflumate |
| Antiinflammatory/antirheumatic agents in combination with corticosteroids | Oxaceprol |
| Antiinflammatory/antirheumatic agents in combination with corticosteroids | Isonixin |
| Antiinflammatory/antirheumatic agents in combination with corticosteroids | Antiinflammatory/antirheumatic agents in combination with corticosteroids |
| Allopurinol | Febuxostat |
| Febuxostat | Febuxostat |
| Colchicine | Colchicine, combinations |
| Colchicine, combinations | Colchicine, combinations |
| Bisphosphonates | Selective estrogen receptor modulators |
| Bisphosphonates | Bisphosphonates |
| Bisphosphonates | Bisphosphonates, combinations |
| Bisphosphonates | Other drugs affecting bone structure and mineralization |
| Bisphosphonates, combinations | Selective estrogen receptor modulators |
| Bisphosphonates, combinations | Bisphosphonates, combinations |
| Bisphosphonates, combinations | Other drugs affecting bone structure and mineralization |
| Other drugs affecting bone structure and mineralization | Selective estrogen receptor modulators |
| Other drugs affecting bone structure and mineralization | Other drugs affecting bone structure and mineralization |
| Opioids in combination with non-opioid analgesics | Opioids in combination with non-opioid analgesics |
| Codeine and paracetamol | Tramadol |
| Codeine and paracetamol | Tapentadol |
| Codeine and paracetamol | Tramadol, combinations |
| Codeine and acetylsalicylic acid | Tramadol |
| Codeine and acetylsalicylic acid | Tapentadol |
| Codeine and acetylsalicylic acid | Tramadol, combinations |
| Codeine and ibuprofen | Tramadol |
| Codeine and ibuprofen | Tapentadol |
| Codeine and ibuprofen | Tramadol, combinations |
| Tramadol and paracetamol | Tapentadol |
| Tramadol and paracetamol | Tramadol, combinations |
| Tramadol and dexketoprofen | Tramadol |
| Tramadol and dexketoprofen | Tapentadol |
| Tramadol and dexketoprofen | Tramadol, combinations |
| Tramadol | Tramadol |
| Tramadol | Tapentadol |
| Tapentadol | Tramadol, combinations |
| Tramadol, combinations | Tramadol, combinations |
| Acetylsalicylic acid | Triflusal |
| Acetylsalicylic acid | Platelet aggregation inhibitors excl. Heparin, combinations |
| Acetylsalicylic acid | Atorvastatin, acetylsalicylic acid and ramipril |
| Acetylsalicylic acid | Acetylsalicylic acid |
| Acetylsalicylic acid | Acetylsalicylic acid, combinations excl. Psycholeptics |
| Acetylsalicylic acid, combinations excl. Psycholeptics | Triflusal |
| Acetylsalicylic acid, combinations excl. Psycholeptics | Platelet aggregation inhibitors excl. Heparin, combinations |
| Acetylsalicylic acid, combinations excl. Psycholeptics | Atorvastatin, acetylsalicylic acid and ramipril |
| Acetylsalicylic acid, combinations excl. Psycholeptics | Acetylsalicylic acid, combinations excl. Psycholeptics |
| Metamizole sodium | Metamizole sodium |
| Paracetamol | Paracetamol |
| Paracetamol, combinations excl. Psycholeptics | Paracetamol, combinations excl. Psycholeptics |
| Paracetamol, combinations excl. Psycholeptics | Tramadol and paracetamol |
| Paracetamol, combinations excl. Psycholeptics | Codeine and paracetamol |
| Tramadol and paracetamol | Tramadol and paracetamol |
| Tramadol and paracetamol | Codeine and paracetamol |
| Codeine and paracetamol | Codeine and paracetamol |
| Selective serotonin (5HT1) agonists | Selective serotonin (5HT1) agonists |
| Phenobarbital | Primidone |
| Primidone | Primidone |
| Diazepam | Ketazolam |
| Diazepam | Diazepam, combinations |
| Diazepam | Flurazepam |
| Diazepam | Quazepam |
| Chlordiazepoxide | Lorazepam |
| Chlordiazepoxide | Halazepam |
| Chlordiazepoxide | Pinazepam |
| Chlordiazepoxide | Flunitrazepam |
| Chlordiazepoxide | Lormetazepam |
| Chlordiazepoxide | Loprazolam |
| Potassium clorazepate | Diazepam |
| Potassium clorazepate | Ketazolam |
| Potassium clorazepate | Diazepam, combinations |
| Potassium clorazepate | Potassium clorazepate, combinations |
| Potassium clorazepate | Flurazepam |
| Potassium clorazepate | Quazepam |
| Lorazepam | Pinazepam |
| Bromazepam | Chlordiazepoxide |
| Bromazepam | Lorazepam |
| Bromazepam | Halazepam |
| Bromazepam | Pinazepam |
| Clobazam | Diazepam |
| Clobazam | Potassium clorazepate |
| Clobazam | Ketazolam |
| Clobazam | Diazepam, combinations |
| Clobazam | Potassium clorazepate, combinations |
| Clobazam | Flurazepam |
| Clobazam | Quazepam |
| Ketazolam | Quazepam |
| Alprazolam | Chlordiazepoxide |
| Alprazolam | Lorazepam |
| Alprazolam | Bromazepam |
| Alprazolam | Alprazolam |
| Alprazolam | Halazepam |
| Alprazolam | Pinazepam |
| Alprazolam | Flunitrazepam |
| Alprazolam | Lormetazepam |
| Alprazolam | Loprazolam |
| Halazepam | Lorazepam |
| Halazepam | Halazepam |
| Halazepam | Pinazepam |
| Halazepam | Lormetazepam |
| Halazepam | Loprazolam |
| Pinazepam | Pinazepam |
| Clotiazepam | Clotiazepam |
| Clotiazepam | Triazolam |
| Clotiazepam | Midazolam |
| Bentazepam | Midazolam |
| Bentazepam | Triazolam |
| Bentazepam | Clotiazepam |
| Bentazepam | Brotizolam |
| Bentazepam | Bentazepam |
| Bentazepam | Clotiazepam |
| Diazepam, combinations | Ketazolam |
| Diazepam, combinations | Diazepam, combinations |
| Diazepam, combinations | Flurazepam |
| Diazepam, combinations | Quazepam |
| Potassium clorazepate, combinations | Diazepam |
| Potassium clorazepate, combinations | Ketazolam |
| Potassium clorazepate, combinations | Diazepam, combinations |
| Potassium clorazepate, combinations | Potassium clorazepate, combinations |
| Potassium clorazepate, combinations | Flurazepam |
| Potassium clorazepate, combinations | Quazepam |
| Bentazepam | Clotiazepam |
| Bentazepam | Bentazepam |
| Bentazepam | Triazolam |
| Bentazepam | Midazolam |
| Bentazepam | Brotizolam |
| Flurazepam | Ketazolam |
| Flurazepam | Flurazepam |
| Flurazepam | Quazepam |
| Flunitrazepam | Lorazepam |
| Flunitrazepam | Halazepam |
| Flunitrazepam | Pinazepam |
| Flunitrazepam | Flunitrazepam |
| Flunitrazepam | Lormetazepam |
| Flunitrazepam | Loprazolam |
| Triazolam | Triazolam |
| Lormetazepam | Pinazepam |
| Midazolam | Triazolam |
| Midazolam | Midazolam |
| Brotizolam | Clotiazepam |
| Brotizolam | Triazolam |
| Brotizolam | Midazolam |
| Brotizolam | Brotizolam |
| Quazepam | Quazepam |
| Loprazolam | Lorazepam |
| Loprazolam | Pinazepam |
| Loprazolam | Lormetazepam |
| Loprazolam | Loprazolam |
| Benzodiazepine related drugs | Benzodiazepine related drugs |
| Imipramine | Trimipramine |
| Imipramine | Nortriptyline |
| Imipramine | Maprotiline |
| Clomipramine | Imipramine |
| Clomipramine | Trimipramine |
| Clomipramine | Nortriptyline |
| Clomipramine | Doxepin |
| Clomipramine | Maprotiline |
| Trimipramine | Trimipramine |
| Nortriptyline | Trimipramine |
| Nortriptyline | Nortriptyline |
| Doxepin | Imipramine |
| Doxepin | Trimipramine |
| Doxepin | Nortriptyline |
| Doxepin | Doxepin |
| Doxepin | Maprotiline |
| Maprotiline | Trimipramine |
| Maprotiline | Nortriptyline |
| Selective serotonin reuptake inhibitors | Selective serotonin reuptake inhibitors |
| Selective serotonin reuptake inhibitors | Vortioxetine |
| Mianserin | Mianserin |
| Mianserin | Trazodone |
| Mianserin | Mirtazapine |
| Trazodone | Trazodone |
| Trazodone | Mirtazapine |
| Mirtazapine | Mirtazapine |
| Venlafaxine | Reboxetine |
| Venlafaxine | Duloxetine |
| Venlafaxine | Desvenlafaxine |
| Reboxetine | Duloxetine |
| Reboxetine | Desvenlafaxine |
| Duloxetine | Desvenlafaxine |
| Salmeterol | Salmeterol |
| Salmeterol | Formoterol |
| Salmeterol | Indacaterol |
| Salmeterol | Olodaterol |
| Salmeterol | Salmeterol and fluticasone |
| Salmeterol | Formoterol and budesonide |
| Salmeterol | Formoterol and beclometasone |
| Salmeterol | Vilanterol and fluticasone furoate |
| Salmeterol | Formoterol and fluticasone |
| Salmeterol | Vilanterol and umeclidinium bromide |
| Salmeterol | Indacaterol and glycopyrronium bromide |
| Salmeterol | Formoterol and aclidinium bromide |
| Salmeterol | Olodaterol and tiotropium bromide |
| Salmeterol | Formoterol and glycopyrronium bromide |
| Salmeterol | Vilanterol, umeclidinium bromide and fluticasone furoate |
| Salmeterol | Formoterol, glycopyrronium bromide and beclometasone |
| Formoterol | Formoterol |
| Formoterol | Indacaterol |
| Formoterol | Olodaterol |
| Formoterol | Salmeterol and fluticasone |
| Formoterol | Formoterol and budesonide |
| Formoterol | Formoterol and beclometasone |
| Formoterol | Vilanterol and fluticasone furoate |
| Formoterol | Formoterol and fluticasone |
| Formoterol | Vilanterol and umeclidinium bromide |
| Formoterol | Indacaterol and glycopyrronium bromide |
| Formoterol | Formoterol and aclidinium bromide |
| Formoterol | Olodaterol and tiotropium bromide |
| Formoterol | Formoterol and glycopyrronium bromide |
| Formoterol | Vilanterol, umeclidinium bromide and fluticasone furoate |
| Formoterol | Formoterol, glycopyrronium bromide and beclometasone |
| Indacaterol | Indacaterol |
| Indacaterol | Olodaterol |
| Indacaterol | Salmeterol and fluticasone |
| Indacaterol | Formoterol and budesonide |
| Indacaterol | Formoterol and beclometasone |
| Indacaterol | Vilanterol and fluticasone furoate |
| Indacaterol | Formoterol and fluticasone |
| Indacaterol | Vilanterol and umeclidinium bromide |
| Indacaterol | Indacaterol and glycopyrronium bromide |
| Indacaterol | Formoterol and aclidinium bromide |
| Indacaterol | Olodaterol and tiotropium bromide |
| Indacaterol | Formoterol and glycopyrronium bromide |
| Indacaterol | Vilanterol, umeclidinium bromide and fluticasone furoate |
| Indacaterol | Formoterol, glycopyrronium bromide and beclometasone |
| Olodaterol | Olodaterol |
| Olodaterol | Salmeterol and fluticasone |
| Olodaterol | Formoterol and budesonide |
| Olodaterol | Formoterol and beclometasone |
| Olodaterol | Vilanterol and fluticasone furoate |
| Olodaterol | Formoterol and fluticasone |
| Olodaterol | Vilanterol and umeclidinium bromide |
| Olodaterol | Indacaterol and glycopyrronium bromide |
| Olodaterol | Formoterol and aclidinium bromide |
| Olodaterol | Olodaterol and tiotropium bromide |
| Olodaterol | Formoterol and glycopyrronium bromide |
| Olodaterol | Vilanterol, umeclidinium bromide and fluticasone furoate |
| Olodaterol | Formoterol, glycopyrronium bromide and beclometasone |
| Salmeterol and fluticasone | Salmeterol and fluticasone |
| Salmeterol and fluticasone | Formoterol and budesonide |
| Salmeterol and fluticasone | Formoterol and beclometasone |
| Salmeterol and fluticasone | Vilanterol and fluticasone furoate |
| Salmeterol and fluticasone | Formoterol and fluticasone |
| Salmeterol and fluticasone | Vilanterol and umeclidinium bromide |
| Salmeterol and fluticasone | Indacaterol and glycopyrronium bromide |
| Salmeterol and fluticasone | Formoterol and aclidinium bromide |
| Salmeterol and fluticasone | Olodaterol and tiotropium bromide |
| Salmeterol and fluticasone | Formoterol and glycopyrronium bromide |
| Salmeterol and fluticasone | Vilanterol, umeclidinium bromide and fluticasone furoate |
| Salmeterol and fluticasone | Formoterol, glycopyrronium bromide and beclometasone |
| Formoterol and budesonide | Formoterol and budesonide |
| Formoterol and budesonide | Formoterol and beclometasone |
| Formoterol and budesonide | Vilanterol and fluticasone furoate |
| Formoterol and budesonide | Formoterol and fluticasone |
| Formoterol and budesonide | Vilanterol and umeclidinium bromide |
| Formoterol and budesonide | Indacaterol and glycopyrronium bromide |
| Formoterol and budesonide | Formoterol and aclidinium bromide |
| Formoterol and budesonide | Olodaterol and tiotropium bromide |
| Formoterol and budesonide | Formoterol and glycopyrronium bromide |
| Formoterol and budesonide | Vilanterol, umeclidinium bromide and fluticasone furoate |
| Formoterol and budesonide | Formoterol, glycopyrronium bromide and beclometasone |
| Formoterol and beclometasone | Formoterol and beclometasone |
| Formoterol and beclometasone | Vilanterol and fluticasone furoate |
| Formoterol and beclometasone | Formoterol and fluticasone |
| Formoterol and beclometasone | Vilanterol and umeclidinium bromide |
| Formoterol and beclometasone | Indacaterol and glycopyrronium bromide |
| Formoterol and beclometasone | Formoterol and aclidinium bromide |
| Formoterol and beclometasone | Olodaterol and tiotropium bromide |
| Formoterol and beclometasone | Formoterol and glycopyrronium bromide |
| Formoterol and beclometasone | Vilanterol, umeclidinium bromide and fluticasone furoate |
| Formoterol and beclometasone | Formoterol, glycopyrronium bromide and beclometasone |
| Vilanterol and fluticasone furoate | Vilanterol and fluticasone furoate |
| Vilanterol and fluticasone furoate | Formoterol and fluticasone |
| Vilanterol and fluticasone furoate | Vilanterol and umeclidinium bromide |
| Vilanterol and fluticasone furoate | Indacaterol and glycopyrronium bromide |
| Vilanterol and fluticasone furoate | Formoterol and aclidinium bromide |
| Vilanterol and fluticasone furoate | Olodaterol and tiotropium bromide |
| Vilanterol and fluticasone furoate | Formoterol and glycopyrronium bromide |
| Vilanterol and fluticasone furoate | Vilanterol, umeclidinium bromide and fluticasone furoate |
| Vilanterol and fluticasone furoate | Formoterol, glycopyrronium bromide and beclometasone |
| Formoterol and fluticasone | Formoterol and fluticasone |
| Formoterol and fluticasone | Vilanterol and umeclidinium bromide |
| Formoterol and fluticasone | Indacaterol and glycopyrronium bromide |
| Formoterol and fluticasone | Formoterol and aclidinium bromide |
| Formoterol and fluticasone | Olodaterol and tiotropium bromide |
| Formoterol and fluticasone | Formoterol and glycopyrronium bromide |
| Formoterol and fluticasone | Vilanterol, umeclidinium bromide and fluticasone furoate |
| Formoterol and fluticasone | Formoterol, glycopyrronium bromide and beclometasone |
| Vilanterol and umeclidinium bromide | Vilanterol and umeclidinium bromide |
| Vilanterol and umeclidinium bromide | Indacaterol and glycopyrronium bromide |
| Vilanterol and umeclidinium bromide | Formoterol and aclidinium bromide |
| Vilanterol and umeclidinium bromide | Olodaterol and tiotropium bromide |
| Vilanterol and umeclidinium bromide | Formoterol and glycopyrronium bromide |
| Vilanterol and umeclidinium bromide | Vilanterol, umeclidinium bromide and fluticasone furoate |
| Vilanterol and umeclidinium bromide | Formoterol, glycopyrronium bromide and beclometasone |
| Indacaterol and glycopyrronium bromide | Indacaterol and glycopyrronium bromide |
| Indacaterol and glycopyrronium bromide | Formoterol and aclidinium bromide |
| Indacaterol and glycopyrronium bromide | Olodaterol and tiotropium bromide |
| Indacaterol and glycopyrronium bromide | Formoterol and glycopyrronium bromide |
| Indacaterol and glycopyrronium bromide | Vilanterol, umeclidinium bromide and fluticasone furoate |
| Indacaterol and glycopyrronium bromide | Formoterol, glycopyrronium bromide and beclometasone |
| Formoterol and aclidinium bromide | Formoterol and aclidinium bromide |
| Formoterol and aclidinium bromide | Olodaterol and tiotropium bromide |
| Formoterol and aclidinium bromide | Formoterol and glycopyrronium bromide |
| Formoterol and aclidinium bromide | Vilanterol, umeclidinium bromide and fluticasone furoate |
| Formoterol and aclidinium bromide | Formoterol, glycopyrronium bromide and beclometasone |
| Olodaterol and tiotropium bromide | Olodaterol and tiotropium bromide |
| Olodaterol and tiotropium bromide | Formoterol and glycopyrronium bromide |
| Olodaterol and tiotropium bromide | Vilanterol, umeclidinium bromide and fluticasone furoate |
| Olodaterol and tiotropium bromide | Formoterol, glycopyrronium bromide and beclometasone |
| Formoterol and glycopyrronium bromide | Formoterol and glycopyrronium bromide |
| Formoterol and glycopyrronium bromide | Vilanterol, umeclidinium bromide and fluticasone furoate |
| Formoterol and glycopyrronium bromide | Formoterol, glycopyrronium bromide and beclometasone |
| Vilanterol, umeclidinium bromide and fluticasone furoate | Vilanterol, umeclidinium bromide and fluticasone furoate |
| Vilanterol, umeclidinium bromide and fluticasone furoate | Formoterol, glycopyrronium bromide and beclometasone |
| Formoterol, glycopyrronium bromide and beclometasone | Formoterol, glycopyrronium bromide and beclometasone |
| Tiotropium bromide | Tiotropium bromide |
| Tiotropium bromide | Aclidinium bromide |
| Tiotropium bromide | Glycopyrronium bromide |
| Tiotropium bromide | Umeclidinium bromide |
| Tiotropium bromide | Vilanterol and umeclidinium bromide |
| Tiotropium bromide | Indacaterol and glycopyrronium bromide |
| Tiotropium bromide | Formoterol and aclidinium bromide |
| Tiotropium bromide | Olodaterol and tiotropium bromide |
| Tiotropium bromide | Formoterol and glycopyrronium bromide |
| Tiotropium bromide | Vilanterol, umeclidinium bromide and fluticasone furoate |
| Tiotropium bromide | Formoterol, glycopyrronium bromide and beclometasone |
| Aclidinium bromide | Aclidinium bromide |
| Aclidinium bromide | Glycopyrronium bromide |
| Aclidinium bromide | Umeclidinium bromide |
| Aclidinium bromide | Vilanterol and umeclidinium bromide |
| Aclidinium bromide | Indacaterol and glycopyrronium bromide |
| Aclidinium bromide | Formoterol and aclidinium bromide |
| Aclidinium bromide | Olodaterol and tiotropium bromide |
| Aclidinium bromide | Formoterol and glycopyrronium bromide |
| Aclidinium bromide | Vilanterol, umeclidinium bromide and fluticasone furoate |
| Aclidinium bromide | Formoterol, glycopyrronium bromide and beclometasone |
| Glycopyrronium bromide | Glycopyrronium bromide |
| Glycopyrronium bromide | Umeclidinium bromide |
| Glycopyrronium bromide | Vilanterol and umeclidinium bromide |
| Glycopyrronium bromide | Indacaterol and glycopyrronium bromide |
| Glycopyrronium bromide | Formoterol and aclidinium bromide |
| Glycopyrronium bromide | Olodaterol and tiotropium bromide |
| Glycopyrronium bromide | Formoterol and glycopyrronium bromide |
| Glycopyrronium bromide | Vilanterol, umeclidinium bromide and fluticasone furoate |
| Glycopyrronium bromide | Formoterol, glycopyrronium bromide and beclometasone |
| Umeclidinium bromide | Umeclidinium bromide |
| Umeclidinium bromide | Vilanterol and umeclidinium bromide |
| Umeclidinium bromide | Indacaterol and glycopyrronium bromide |
| Umeclidinium bromide | Formoterol and aclidinium bromide |
| Umeclidinium bromide | Olodaterol and tiotropium bromide |
| Umeclidinium bromide | Formoterol and glycopyrronium bromide |
| Umeclidinium bromide | Vilanterol, umeclidinium bromide and fluticasone furoate |
| Umeclidinium bromide | Formoterol, glycopyrronium bromide and beclometasone |
| Vilanterol and umeclidinium bromide | Vilanterol and umeclidinium bromide |
| Vilanterol and umeclidinium bromide | Indacaterol and glycopyrronium bromide |
| Vilanterol and umeclidinium bromide | Formoterol and aclidinium bromide |
| Vilanterol and umeclidinium bromide | Olodaterol and tiotropium bromide |
| Vilanterol and umeclidinium bromide | Formoterol and glycopyrronium bromide |
| Vilanterol and umeclidinium bromide | Vilanterol, umeclidinium bromide and fluticasone furoate |
| Vilanterol and umeclidinium bromide | Formoterol, glycopyrronium bromide and beclometasone |
| Indacaterol and glycopyrronium bromide | Indacaterol and glycopyrronium bromide |
| Indacaterol and glycopyrronium bromide | Formoterol and aclidinium bromide |
| Indacaterol and glycopyrronium bromide | Olodaterol and tiotropium bromide |
| Indacaterol and glycopyrronium bromide | Formoterol and glycopyrronium bromide |
| Indacaterol and glycopyrronium bromide | Vilanterol, umeclidinium bromide and fluticasone furoate |
| Indacaterol and glycopyrronium bromide | Formoterol, glycopyrronium bromide and beclometasone |
| Formoterol and aclidinium bromide | Formoterol and aclidinium bromide |
| Formoterol and aclidinium bromide | Olodaterol and tiotropium bromide |
| Formoterol and aclidinium bromide | Formoterol and glycopyrronium bromide |
| Formoterol and aclidinium bromide | Vilanterol, umeclidinium bromide and fluticasone furoate |
| Formoterol and aclidinium bromide | Formoterol, glycopyrronium bromide and beclometasone |
| Olodaterol and tiotropium bromide | Olodaterol and tiotropium bromide |
| Olodaterol and tiotropium bromide | Formoterol and glycopyrronium bromide |
| Olodaterol and tiotropium bromide | Vilanterol, umeclidinium bromide and fluticasone furoate |
| Olodaterol and tiotropium bromide | Formoterol, glycopyrronium bromide and beclometasone |
| Formoterol and glycopyrronium bromide | Formoterol and glycopyrronium bromide |
| Formoterol and glycopyrronium bromide | Vilanterol, umeclidinium bromide and fluticasone furoate |
| Formoterol and glycopyrronium bromide | Formoterol, glycopyrronium bromide and beclometasone |
| Vilanterol, umeclidinium bromide and fluticasone furoate | Vilanterol, umeclidinium bromide and fluticasone furoate |
| Vilanterol, umeclidinium bromide and fluticasone furoate | Formoterol, glycopyrronium bromide and beclometasone |
| Formoterol, glycopyrronium bromide and beclometasone | Formoterol, glycopyrronium bromide and beclometasone |
| Beclometasone | Beclometasone |
| Beclometasone | Budesonide |
| Beclometasone | Fluticasone |
| Beclometasone | Mometasone |
| Beclometasone | Ciclesonide |
| Beclometasone | Salmeterol and fluticasone |
| Beclometasone | Formoterol and budesonide |
| Beclometasone | Formoterol and mometasone |
| Beclometasone | Vilanterol and fluticasone furoate |
| Beclometasone | Formoterol and fluticasone |
| Budesonide | Budesonide |
| Budesonide | Fluticasone |
| Budesonide | Mometasone |
| Budesonide | Ciclesonide |
| Budesonide | Salmeterol and fluticasone |
| Budesonide | Formoterol and beclometasone |
| Budesonide | Formoterol and mometasone |
| Budesonide | Vilanterol and fluticasone furoate |
| Budesonide | Formoterol and fluticasone |
| Fluticasone | Fluticasone |
| Fluticasone | Mometasone |
| Fluticasone | Ciclesonide |
| Fluticasone | Formoterol and budesonide |
| Fluticasone | Formoterol and beclometasone |
| Fluticasone | Formoterol and mometasone |
| Mometasone | Mometasone |
| Mometasone | Ciclesonide |
| Mometasone | Salmeterol and fluticasone |
| Mometasone | Formoterol and budesonide |
| Mometasone | Formoterol and beclometasone |
| Mometasone | Vilanterol and fluticasone furoate |
| Mometasone | Formoterol and fluticasone |
| Ciclesonide | Ciclesonide |
| Ciclesonide | Salmeterol and fluticasone |
| Ciclesonide | Formoterol and budesonide |
| Ciclesonide | Formoterol and beclometasone |
| Ciclesonide | Formoterol and mometasone |
| Ciclesonide | Vilanterol and fluticasone furoate |
| Ciclesonide | Formoterol and fluticasone |
| Salmeterol and fluticasone | Salmeterol and fluticasone |
| Salmeterol and fluticasone | Formoterol and budesonide |
| Salmeterol and fluticasone | Formoterol and beclometasone |
| Salmeterol and fluticasone | Formoterol and mometasone |
| Salmeterol and fluticasone | Vilanterol and fluticasone furoate |
| Salmeterol and fluticasone | Formoterol and fluticasone |
| Formoterol and budesonide | Formoterol and budesonide |
| Formoterol and budesonide | Formoterol and beclometasone |
| Formoterol and budesonide | Formoterol and mometasone |
| Formoterol and budesonide | Vilanterol and fluticasone furoate |
| Formoterol and budesonide | Formoterol and fluticasone |
| Formoterol and beclometasone | Formoterol and beclometasone |
| Formoterol and beclometasone | Formoterol and mometasone |
| Formoterol and beclometasone | Vilanterol and fluticasone furoate |
| Formoterol and beclometasone | Formoterol and fluticasone |
| Formoterol and mometasone | Formoterol and mometasone |
| Formoterol and mometasone | Vilanterol and fluticasone furoate |
| Formoterol and mometasone | Formoterol and fluticasone |
| Vilanterol and fluticasone furoate | Vilanterol and fluticasone furoate |
| Vilanterol and fluticasone furoate | Formoterol and fluticasone |
| Formoterol and fluticasone | Formoterol and fluticasone |

ACE inhibitors: Angiotensin-converting enzyme inhibitors; HMG CoA reductase inhibitors: Hydroxy-methylglutaryl coenzyme A reductase inhibitors

**Table S4. List of drug-drug interaction†**

| **Therapeutic group/Drug A** | **Therapeutic group/Drug B** |
| --- | --- |
| Aprepitant | Pimozide |
| Dabigatran etexilate | Dronedarone |
| Dabigatran etexilate | Tacrolimus |
| Dabigatran etexilate | Itraconazole |
| Dronedarone | CYP3A4 inhibitors |
| Ivabradine | CYP3A4 inhibitors |
| Ranolazine | CYP3A4 inhibitors |
| Potassium-sparing agents | Potassium |
| Diuretics and potassium-sparing agents in combination | Potassium |
| Diltiazem | Sertindole |
| Aliskiren | Ciclosporin |
| Aliskiren | Itraconazole |
| Simvastatin | Itraconazole |
| Lipid modifying agents, combinations | Itraconazole |
| Simvastatin | Telithromycin |
| Lipid modifying agents, combinations | Telithromycin |
| Lovastatin | Itraconazole |
| Lovastatin | Telithromycin |
| Atorvastatin | Itraconazole |
| Atorvastatin | Telithromycin |
| Atorvastatin and amlodipine | Itraconazole |
| Atorvastatin and amlodipine | Telithromycin |
| Gemfibrozil | Repaglinide |
| Fibrates | Rosuvastatin |
| HMG CoA reductase inhibitors | Fusidic acid |
| HMG CoA reductase inhibitors in combination with other lipid modifying agents | Fusidic acid |
| HMG CoA reductase inhibitors, other combinations | Fusidic acid |
| CYP3A4 inhibitors | Pimozide |
| Oral retinoids | Tetracyclines |
| Oral retinoids | Vitamin A, plain |
| Dapoxetine | CYP3A4 inhibitors |
| Erythromycin | Mizolastine |
| Erythromycin | Sertindole |
| Erythromycin | Simvastatin |
| Erythromycin | Lipid modifying agents, combinations |
| Clarithromycin | Mizolastine |
| Clarithromycin | Sertindole |
| Clarithromycin | Simvastatin |
| Clarithromycin | Lipid modifying agents, combinations |
| Clarithromycin | Ticagrelor |
| Fluconazole | Pimozide |
| Itraconazole | Triazolam |
| Itraconazole | Sertindole |
| Itraconazole | Mizolastine |
| Methotrexate | Trimethoprim |
| Ciclosporin | Dabigatran etexilate |
| Ciclosporin | Rosuvastatin |
| Preparations inhibiting uric acid production | Azathioprine |
| Colchicine | Verapamil |
| Colchicine | Clarithromycin |
| Colchicine | Erythromycin |
| Colchicine | Telithromycin |
| Naltrexone | Methadone |
| Nalmefene | Methadone |
| Sumatriptan | Monoamine oxidase inhibitors |
| Zolmitriptan | Monoamine oxidase inhibitors |
| Rizatriptan | Monoamine oxidase inhibitors |
| Almotriptan | Monoamine oxidase inhibitors |
| Ergot alkaloids | Clarithromycin |
| Ergot alkaloids | Erythromycin |
| Ergot alkaloids | Itraconazole |
| Ergot alkaloids | Telithromycin |
| Ergot alkaloids | Selective serotonin (5HT1) agonists |
| Entacapone | Monoamine oxidase inhibitors, non-selective |
| Sertindole | Verapamil |
| Pimozide | Sertraline |
| Pimozide | Verapamil |
| Paroxetine | Pimozide |
| Monoamine oxidase inhibitors, non-selective | Selective serotonin reuptake inhibitors |
| Monoamine oxidase inhibitors, non-selective | Adrenergics and serotonergic drugs |
| Monoamine oxidase inhibitors, non-selective | Indirect sympathomimetics |
| Monoamine oxidase A inhibitors | Monoamine oxidase B inhibitors |
| Duloxetine | Fluvoxamine |
| Agomelatine | Fluvoxamine |
| Monoamine oxidase B inhibitors | Bupropion |
| Monoamine oxidase A inhibitors | Dextromethorphan |
| Monoamine oxidase inhibitors, non-selective | Dextromethorphan |
| Monoamine oxidase inhibitors, non-selective | Tetrabenazine |
| Monoamine oxidase A inhibitors | Tramadol |
| Monoamine oxidase inhibitors, non-selective | Tramadol |
| Monoamine oxidase inhibitors | Pethidine |
| Brivudine | Fluorouracil |
| Brivudine | Fluorouracil, combinations |
| Brivudine | Tegafur |
| Brivudine | Tegafur, combinations |
| Drug induced TdP (excl. citalopram, escitalopram, domperidone, hydroxyzine, methadone , antipsychotics and antiparasitic products inductors of TdP) | Drug induced TdP (excl. citalopram, escitalopram, domperidone, hydroxyzine, methadone , antipsychotics and antiparasitic products inductors of TdP) |
| Drug induced TdP (excl. citalopram, escitalopram, domperidone, hydroxyzine, methadone , antipsychotics and antiparasitic products inductors of TdP) | Domperidone |
| Drug induced TdP (excl. citalopram, escitalopram, domperidone, hydroxyzine, methadone , antipsychotics and antiparasitic products inductors of TdP) | Hydroxyzine |
| Drug induced TdP (excl. citalopram, escitalopram, domperidone, hydroxyzine, methadone , antipsychotics and antiparasitic products inductors of TdP) | Citalopram |
| Drug induced TdP (excl. citalopram, escitalopram, domperidone, hydroxyzine, methadone , antipsychotics and antiparasitic products inductors of TdP) | Escitalopram |
| Antipsychotics inductors of TdP | Domperidone |
| Antipsychotics inductors of TdP | Hydroxyzine |
| Antipsychotics inductors of TdP | Citalopram |
| Antipsychotics inductors of TdP | Escitalopram |
| Antiparasitic products inductors of TdP | Domperidone |
| Antiparasitic products inductors of TdP | Hydroxyzine |
| Antiparasitic products inductors of TdP | Citalopram |
| Antiparasitic products inductors of TdP | Escitalopram |
| Domperidone | Hydroxyzine |
| Domperidone | Citalopram |
| Domperidone | Escitalopram |
| Hydroxyzine | Citalopram |
| Hydroxyzine | Escitalopram |
| Citalopram | Escitalopram |
| Methadone | Domperidone |
| Methadone | Hydroxyzine |
| Methadone | Citalopram |
| Methadone | Escitalopram |
| Valsartan and sacubitril | ACE inhibitors, plain |
| Valsartan and sacubitril | Angiotensin II receptor blockers, plain |
| Valsartan and sacubitril | Renin-inhibitors |
| Angiotensin II receptor blockers, plain | Renin-inhibitors |
| Angiotensin II receptor blockers, combinations | Renin-inhibitors |
| ACE inhibitors, plain | Renin-inhibitors |
| ACE inhibitors, combinations | Renin-inhibitors |

ACE inhibitors: Angiotensin-converting enzyme inhibidors; HMG CoA reductase inhibidors: Hydroxy-methylglutaryl coenzyme A reductase inhibitors; TdP: Torsades de pointes

†References

1. Agence nationale de sécurité du médicament et des produits de santé (ANSM). Thesaurus des interactions médicamenteuses. 2018. 1–251 p.
2. Joint Formulary Committee. British National Formulary (BNF) [Internet]. [cited 2019 Nov 30]. Available from: <https://about.medicinescomplete.com/publication/british-national-formulary/>
3. Lexicomp® Drug Interactions - UpToDate [Internet]. 2018 [cited 2018 Nov 27]. Available from: <https://146.219.19.12/drug-interactions/?source=responsive_home#di-disclaimer>

**Table S5. List of drugs contraindicated in chronic kidney disease by first level of ATC**†

| **Anatomical/pharmacological group** | **Therapeutic group** | **Drug** | **Condition** |
| --- | --- | --- | --- |
| A: Alimentary tract and metabolism | Drugs for acid related disorders | Magnesium hydroxide | GFR < 30 ml/min/1,73m^2^ |
|  |  | Algedrate/Magnesium hydroxide |  |
|  |  | Algedrate/Magnesium carbonate/Magnesium hydroxide |  |
|  |  | Sucralfate |  |
|  |  | Almagate | GFR < 10 ml/min/1,73m^2^ |
|  | Antidiarrheals, intestinal antiinflammatory/antiinfective agents | Sulfasalazina | GFR < 50 ml/min/1,73m^2^ |
|  | Drugs used in diabetes | Saxagliptin | Dialysis |
|  |  | Metformin | GFR < 30 ml/min/1,73m^2^ |
|  |  | Glipizide |  |
|  |  | Gliclazide |  |
|  |  | Glimepiride |  |
|  |  | Glysentide |  |
|  |  | Metformin/pioglitazone |  |
|  |  | Glimepiride/pioglitazone |  |
|  |  | Metformin/sitagliptin |  |
|  |  | Meformin/vildagliptin |  |
|  |  | Metformin/saxagliptin |  |
|  |  | Metformin/linagliptin |  |
|  |  | Metformin/alogliptin |  |
|  |  | Acarbose |  |
|  |  | Miglitol |  |
|  |  | Exenatide |  |
|  |  | Liraglutide |  |
|  |  | Lixisenatide |  |
|  |  | Albiglutide |  |
|  |  | Dulaglutide |  |
|  |  | Exenatide |  |
|  |  | Liraglutide |  |
|  |  | Albiglutide |  |
|  |  | Dulaglutide |  |
|  |  | Canaglifozin/metformin | GFR < 45 ml/min/1,73m^2^ |
|  |  | Empaglifozin/metformin |  |
|  |  | Canaglifozin |  |
|  |  | Empaglifozin |  |
|  |  | Canaglifozin |  |
|  |  | Empaglifozin |  |
|  |  | Glibenclamide | GFR < 60 ml/min/1,73m^2^ |
|  |  | Dapagliflozin/metformin |  |
|  |  | Dapaglifozin |  |
|  | Mineral supplements | Potassium | GFR < 30 ml/min/1,73m^2^ |
| B: Blood and blood forming organs | Antithrombotic agents | Tinzaparin | GFR < 10 ml/min/1,73m^2^ |
|  |  | Rivaroxaban |  |
|  |  | Apixaban | GFR < 15 ml/min/1,73m^2^ |
|  |  | Edoxaban |  |
|  |  | Cilostazol | GFR < 25 ml/min/1,73m^2^ |
|  |  | Nadroparin | GFR < 30 ml/min/1,73m^2^ |
|  |  | Dabigatran etexilate |  |
|  |  | Fondaparinux |  |
| C: Cardiovascular system | Cardiac therapy | Trimetazidine | GFR < 30 ml/min/1,73m^2^ |
|  |  | Ranolazine |  |
|  | Diuretics | Amiloride | GFR < 10 ml/min/1,73m^2^ |
|  |  | Furosemide and potassium-sparing agents | GFR < 20 ml/min/1,73m^2^ |
|  |  | Hydrochlorothiazide | GFR < 30 ml/min/1,73m^2^ |
|  |  | Sulfonamides, plain |  |
|  |  | Aldosterone antagonists |  |
|  |  | Low-ceiling diuretics and potassium-sparing agents |  |
|  | Beta blocking agents | Beta blocking agents, selective, and thiazides | GFR < 30 ml/min/1,73m^2^ |
|  |  | Beta blocking agents, non-selective, and other diuretics |  |
|  |  | Beta blocking agents, selective, and other diuretics |  |
|  |  | Beta blocking agents, selective, thiazides and other diuretics |  |
|  | Calcium channel blockers | Manidipine | GFR < 10 ml/min/1,73m^2^ |
|  |  | Barnidipine |  |
|  |  | Lercanidipine | GFR < 30 ml/min/1,73m^2^ |
|  | Agents acting on the renin-angiotensin system | Quinapril | GFR < 10 ml/min/1,73m^2^ |
|  |  | Delapril/manidipine |  |
|  |  | Eprosartan |  |
|  |  | Valsartan |  |
|  |  | Valsartan/amlodipine |  |
|  |  | Olmesartan medoxomil | GFR < 20 ml/min/1,73m^2^ |
|  |  | Olmesartan/amlodipine |  |
|  |  | ACE inhibitors and diuretics | GFR < 30 ml/min/1,73m^2^ |
|  |  | Enalapril / lercanidipine |  |
|  |  | Angiotensin II antagonists and diuretics |  |
|  |  | Valsartan/amlodipine/hydrochlorothiazide |  |
|  |  | Olmesartan/amlodipine/hydrochlorothiazide |  |
|  |  | Valsartan/sacubitril |  |
|  |  | Aliskiren |  |
|  |  | Aliskiren/hydrochlorothiazide |  |
|  | Lipid modifying agents | Genfibrozil | GFR < 10 ml/min/1,73m^2^ |
|  |  | Bezafibrate | GFR < 15 ml/min/1,73m^2^ |
|  |  | Rosuvastatine | GFR < 30 ml/min/1,73m^2^ |
|  |  | Fenofibrate |  |
|  |  | Pravastatin/fenofibrate | GFR < 60 ml/min/1,73m^2^ |
| D: Dermatologicals | Antipsoriatics | Acitretin | GFR < 30 ml/min/1,73m^2^ |
| G: Genitorurinary system and sex hormones | Sex hormones and modulators of the genital system | Raloxifene | GFR < 30 ml/min/1,73m^2^ |
|  | Urologicals | Silodosin | GFR < 30 ml/min/1,73m^2^ |
| H: Systemic hormonal preparations, excl. sex hormones and insulins | Pituitary and hypothalamic hormones and analogues | Cetrorelix | GFR < 30 ml/min/1,73m^2^ |
|  | Calcium homeostasis | Teriparatide | GFR < 30 ml/min/1,73m^2^ |
| J: Antiinfectives for systemic use | Antibacterials for systemic use | Trimethoprim | Dialisi |
|  |  | Tetracycline | GFR < 10 ml/min/1,73m^2^ |
|  |  | Sulfamethoxazole/trimethoprim |  |
|  |  | Fosfomycin |  |
|  |  | Nitrofurantoin | GFR < 50 ml/min/1,73m^2^ |
|  | Antimycobacterials | Pyrazinamide | Dialysis |
|  |  | Rifampicin/isoniazid | GFR < 25 ml/min/1,73m^2^ |
|  |  | Rifampicin/pyrazinamide/isoniazid |  |
|  |  | Rifampicin/pyrazinamidy/ethambutol/isoniazida |  |
|  | Antivirals for systemic use | Valganciclovir | GFR < 10 ml/min/1,73m^2^ |
| L: Antineoplastic and immunomodulating agents | Antineoplastic agents | Methotrexate | GFR < 10 ml/min/1,73m^2^ |
|  | Immunosuppressants |  |  |
| M: Musculo-skeletal system | Antiinflammatory and antirheumatic products | Diclofenac | GFR < 30 ml/min/1,73m^2^ |
|  |  | Diclofenac/misoprostol |  |
|  |  | Ibuprofen |  |
|  |  | Naproxen |  |
|  |  | Esomeprazole/naproxen |  |
|  |  | Celecoxib |  |
|  | Antigout preparations | Febuxostat | GFR < 30 ml/min/1,73m^2^ |
|  |  | Colchicine |  |
|  |  | Colchicine/dicycloverine |  |
|  | Drugs for treatment of bone diseases | Bisphosphonates | GFR < 30 ml/min/1,73m^2^ |
|  |  | Bisphosphonates, combinations |  |
|  |  | Strontium ranelate |  |
| N: Nervous system | Analgesics | Oxycodone | GFR < 10 ml/min/1,73m^2^ |
|  |  | Oxycodone/naloxone |  |
|  |  | Codeine/acetylsalicylic acid |  |
|  |  | Acetylsalicylic acid |  |
|  |  | Acetylsalicylic acid, combinations excl. psycholeptics |  |
|  |  | Tramadol retard | GFR < 30 ml/min/1,73m^2^ |
|  |  | Ergot alkaloids |  |
|  | Antiepileptics | Eslicarbazepine | GFR < 30 ml/min/1,73m^2^ |
|  | Anti-parkinson drugs | Ropinirole | GFR < 30 ml/min/1,73m^2^ |
|  | Psycholeptics | Lithium | GFR < 10 ml/min/1,73m^2^ |
|  |  | Hydroxyzine |  |
|  | Psychoanaleptics | Citalopram | GFR < 30 ml/min/1,73m^2^ |
|  |  | Duloxetine |  |
|  |  | Galantamine |  |
|  | Other nervous system drugs | Acamprosate | GFR < 30 ml/min/1,73m^2^ |
| R: Respiratory system | Antihistamines for systemic use | Cetirizine | GFR < 10 ml/min/1,73m^2^ |
|  |  | Levocetirizine |  |
|  |  | Cetirizine/pseudoephedrine |  |
| S: Sensory organs | Ophthalmologicals | Acetazolamide | GFR < 10 ml/min/1,73m^2^ |

ACE inhibitors: Angiotensin-converting enzyme inhibidors; GFR: Glomerular filtration rate

†References

1. Programa d’Harmonització en l’Àmbit de l’Atenció Primària i Comunitària del Servei Català de la Salut. Pautes per a l’harmonització del tractament farmacològic de la malaltia renal crònica [Internet]. Barcelona; 2016. Available from: <http://catsalut.gencat.cat/ca/detalls/articles/malaltia-renal-cronica>

**Table S6. List of drugs contraindicated in liver diseases by first level of ATC**†

| **Anatomical/pharmacological group** | **Therapeutic groups** | **Drug** |
| --- | --- | --- |
| A: Alimentary tract and metabolism | Bile and liver therapy | Ursodeoxycholic acid |
|  | Antidiarrheals, intestinal antiinflammatory/antiinfective agents | Budesonide |
|  | Antiobesity preparations, excl. diet products | Orlistat |
|  | Drugs used in diabetes | Metformin  Metformin/sitagliptin  Meformin/vildagliptin  Metformin/saxagliptin  Metformin/linagliptin  Metformin/alogliptin  Dapagliflozin/metformin  Canaglifozin/metformin  Empaglifozin/metformin |
|  |  | Metformin/pioglitazone |
|  |  | Glimepiride/pioglitazone |
|  |  | Pioglitazone |
|  |  | Alpha glucosidase inhibitors |
|  |  | Repaglinide |
|  |  | Nateglinide |
|  | Vitamins | Pyridoxal phosphate |
| B: Blood and blood forming organs | Antianemic preparations | Ferrimanitol ovoalbumin |
| C: Cardiovascular system | Cardiac therapy | Ranolazine |
|  | Diuretics | Thiazides, plain |
|  |  | Sulfonamides, plain |
|  |  | Hydrochlorothiazide/potassium-sparing agents |
|  | Beta blocking agents | Nebivolol |
|  |  | Atenolol/thiazides |
|  |  | Bisoprolol / thiazides |
|  |  | Nebivolol / thiazides |
|  |  | Atenolol/thiazides/other diuretics |
|  | Calcium channel blockers | Manidipine |
|  |  | Barnidipine |
|  | Agents acting on the renin-angiotensin system | ACE inhibitors and diuretics |
|  |  | Angiotensin II antagonists, plain |
|  |  | Angiotensin II antagonists and diuretics |
|  |  | Angiotensin II antagonists, other combinations |
|  |  | Aliskiren / hydrochlorothiazide |
|  |  | Aliskiren / amlodipine / hydrochlorothiazide |
|  | Lipid modifying agents | Statins |
|  |  | Fibrates |
|  |  | Colestipol |
|  |  | Colesevelam |
|  |  | Ezetimibe |
|  |  | HMG CoA reductase inhibitors in combination with other lipid modifying agents |
|  |  | Atorvastatin / acetylsalicylic acid / ramipril |
|  |  | Nicotinic acid and derivatives |
| D: Dermatologicals | Antipsoriatics | Calcitriol |
|  |  | Acitretin |
|  | Antibiotics and chemotherapeutics for dermatological use | Sinecatechins |
|  | Anti-acne preparations | Isotretinoin |
|  | Other dermatological preparations | Alitretinoin |
| G: Genito urinary system and sex hormones | Other gynecologicals | Cabergoline (gynaecological) |
|  | Sex hormones and modulators of the genital system | Etonogestrel |
|  |  | Estradiol |
|  |  | Estriol |
|  |  | Promestriene |
|  |  | Conjugated estrogens |
|  |  | Tibolone |
|  |  | Medroxyprogesterone |
|  |  | Progesterone |
|  |  | Norethisterone |
|  |  | Clomifene |
|  |  | Cyproterone |
|  |  | Raloxifene |
|  | Urologicals | Acetohydroxamic acid |
| H: Systemic hormonal preparations, excl. sex hormones and insulins | Pituitary and hypothalamic hormones and analogues | Carbetocin |
| J: Anti-infective for systemic use | Antibacterials for systemic use | Telithromycin |
|  |  | Moxifloxacin |
| L: Antineoplastic and immunomdulating agents | Immunosuppressants | Leflunomide |
|  |  | Metotrexate (immunosuppressant) |
| M: Musculo-skeletal system | Antiinflammatory and antirheumatic products | Diacerein |
| N: Nervous system | Analgesics | Hydromorphone |
|  | Antiepileptics | Clonazepam |
|  |  | Valproic, acid |
|  |  | Stiripentol |
|  | Anti-parkinson drugs | Apomorphine |
|  |  | Tolcapone |
|  |  | Entacapone |
|  | Psycholeptics | Chlorpromazine |
|  |  | Levomepromazine |
|  |  | Fluphenazine |
|  |  | Perphenazine |
|  |  | Periciazine |
|  |  | Haloperidol |
|  |  | Pimozide |
|  |  | Clozapine |
|  |  | Clotiapine |
|  |  | Oxazepam |
|  |  | Triazolam |
|  |  | Lormetazepam |
|  |  | Midazolam |
|  |  | Zopiclone |
|  |  | Zolpidem |
|  | Psychoanaleptics | Bupropion |
|  |  | Duloxetine |
|  |  | Agomelatine |
| P: Antiparasitic products, insectcides and repellents | Antiprotozoals | Meglumine antimonate |
| R: Respiratory system | Drugs for obstructive airway diseases | Roflumilast |
|  |  | Zafirlukast |
| S: Sensory organs | Ophthalmologicals | Acetazolamide |

ACE inhibitors: Angiotensin-converting enzyme inhibitors; HMG CoA reductase inhibitors: Hydroxy-methylglutaryl coenzyme A reductase inhibitors

†References

1. Botplus Web [database on the Internet]. Consejo General de Colegios Oficiales de Farmacéuticos. Available from: <https://botplusweb.portalfarma.com>

**Table S7. List of drugs potentially inappropriate in the elderly (≥65 years)** **†**

**Drugs with anticholinergic effect-anticholinergic load**

| **Group therapeutic** | **Drug** | **Anticholinergic load** |
| --- | --- | --- |
| A: Alimentary tract and metabolism | Ranitidine | 1 |
|  | Famotidine | 1 |
|  | Atropine | 2 |
|  | Butylscopolamine | 2 |
|  | Butylscopolamine and analgesics | 2 |
|  | Domperidone | 1 |
|  | Scopolamine | 2 |
|  | Loperamide | 1 |
|  | Loperamide, combinations | 1 |
| C: Cardiovascular system | Disopyramide | 1 |
| G: Genito urinary system and sex hormones | Flavoxate | 2 |
|  | Oxybutynin | 2 |
|  | Propiverine | 2 |
|  | Tolterodine | 2 |
|  | Solifenacin | 2 |
|  | Trospium | 2 |
|  | Fesoterodine | 2 |
|  | Tamsulosin and solifenacin | 2 |
| M: Musculo-skeletal system | Ketorolac | 1 |
|  | Methocarbamol | 1 |
|  | Methocarbamol, combinations excl. psycholeptics | 1 |
|  | Baclofen | 1 |
|  | Tizanidine | 2 |
|  | Cyclobenzaprine | 1 |
| N: Nervous system | Morphine | 1 |
|  | Oxycodone | 1 |
|  | Oxycodone and naloxone | 1 |
|  | Codeine, combinations with psycholeptics | 1 |
|  | Pethidine | 1 |
|  | Fentanyl | 1 |
|  | Dextropropoxyphene | 1 |
|  | Codeine and paracetamol | 1 |
|  | Codeine and acetylsalicylic acid | 1 |
|  | Codeine and ibuprofen | 1 |
|  | Tramadol and paracetamol | 1 |
|  | Tramadol and dexketoprofen | 1 |
|  | Tramadol | 1 |
|  | Clonazepam | 1 |
|  | Carbamazepine | 1 |
|  | Oxcarbamazepine | 1 |
|  | Trihexyphenidyl | 2 |
|  | Biperiden | 2 |
|  | Procyclidine | 2 |
|  | Levodopa, decarboxylase inhibitor and COMT inhibitor | 1 |
|  | Bromocriptine | 1 |
|  | Entacapone | 1 |
|  | Chlorpromazine | 2 |
|  | Levomepromazine | 2 |
|  | Fluphenazine | 2 |
|  | Perphenazine | 1 |
|  | Haloperidol | 1 |
|  | Pimozide | 1 |
|  | Loxapine | 1 |
|  | Clozapine | 2 |
|  | Olanzapine | 1 |
|  | Quetiapine | 1 |
|  | Lithium | 1 |
|  | Risperidone | 1 |
|  | Diazepam | 1 |
|  | Chlordiazepoxide | 1 |
|  | Diazepam, combinations | 1 |
|  | Hydroxyzine | 2 |
|  | Triazolam | 1 |
|  | Imipramine | 2 |
|  | Clomipramine | 2 |
|  | Trimipramine | 2 |
|  | Amitriptyline | 2 |
|  | Nortriptyline | 2 |
|  | Doxepin | 2 |
|  | Fluoxetine | 1 |
|  | Paroxetine | 2 |
|  | Citalopram | 1 |
|  | Fluvoxamine | 1 |
|  | Escitalopram | 1 |
|  | Trazodone | 1 |
|  | Mirtazapine | 1 |
|  | Amitriptyline and psycholeptics | 2 |
|  | Methadone | 1 |
| R: Respiratory system | Pseudoephedrine, combinations | 1 |
|  | Theophylline | 1 |
|  | Codeine | 1 |
|  | Diphenhydramine | 2 |
|  | Doxylamine | 2 |
|  | Diphenhydramine, combinations | 2 |
|  | Doxylamine, combinations | 2 |
|  | Dexchlorpheniramine | 2 |
|  | Alimemazine | 1 |
|  | Mequitazine | 1 |
|  | Promethazine | 2 |
|  | Meclozine | 2 |
|  | Cetirizine | 1 |
|  | Levocetiricine | 1 |
|  | Cyproheptadine | 2 |
|  | Loratadine | 1 |
|  | Ketotifen | 1 |
|  | Ebastine | 1 |
|  | Mizolastine | 1 |
|  | Fexofenadine | 1 |
|  | Desloratidine | 1 |
|  | Rupatadine | 1 |
|  | Bilastine | 1 |
| S: Sensory organs | Cyclopentolate | 2 |

COMT inhibitor: Catechol-O-methyl transferase inhibitor

**Drugs and risk of fall**

| **Group therapeutic** | **Drug** |
| --- | --- |
| C: Cardiovascular system | Methyldopa (levorotatory) |
|  | Clonidine |
|  | Moxonidine |
|  | Prazosin |
|  | Doxazosin |
|  | Hydralazine |
| G: Genito urinary system and sex hormones | Oxybutynin |
|  | Tolterodine |
|  | Solifenacin |
|  | Trospium |
|  | Fesoterodine |
|  | Alfuzosin |
|  | Tamsulosin |
|  | Terazosin |
|  | Silodosin |
|  | Tamsulosin and dutasteride |
| J: Anti-infective for systemic use | Amantadine |
| M: Musculo-skeletal system | Baclofen |
|  | Tizanidine |
|  | Cyclobenzaprine |
| N: Nervous system | Opioids |
|  | Clonazepam |
|  | Carbamazepine |
|  | Oxcarbamazepine |
|  | Trihexyphenidyl |
|  | Biperiden |
|  | Procyclidine |
|  | Chlorpromazine |
|  | Levomepromazine |
|  | Fluphenazine |
|  | Perphenazine |
|  | Periciazine |
|  | Haloperidol |
|  | Sertindole |
|  | Ziprasidone |
|  | Zuclopenthixol |
|  | Pimozide |
|  | Clozapine |
|  | Olanzapine |
|  | Quetiapine |
|  | Asenapine |
|  | Clotiapine |
|  | Sulpiride |
|  | Tiapride |
|  | Amisulpride |
|  | Sulpiride, combinations |
|  | Lithium |
|  | Risperidone |
|  | Aripiprazole |
|  | Paliperidone |
|  | Benzodiazepine derivatives |
|  | Hydroxyzine |
|  | Benzodiazepine derivatives |
|  | Benzodiazepine related drugs |
|  | Imipramine |
|  | Clomipramine |
|  | Trimipramine |
|  | Amitriptyline |
|  | Nortriptyline |
|  | Doxepin |
| R: Respiratory system | Dexchlorpheniramine |
|  | Cyproheptadine |

**Drugs that affect QT interval**

| **Group therapeutic** | **Drug** |
| --- | --- |
| A: Alimentary tract and metabolism | Domperidone |
|  | Ondansetron |
| B: Blood and blood forming organs | Cilostazol |
| C: Cardiovascular system | Procainamide |
|  | Disopyramide |
|  | Flecainide |
|  | Amiodarone |
|  | Dronedarone |
|  | Sotalol |
| H: Systemic hormonal preparations, excl. sex hormones and insulins | Terlipressin |
| J: Anti-infective for systemic use | Erythromycin |
|  | Roxithromycin |
|  | Clarithromycin |
|  | Azithromycin |
|  | Ciprofloxacin |
|  | Levofloxacin |
|  | Moxifloxacin |
|  | Fluconazole |
| L: Antineoplastic and immunomdulating agents | Anagrelide |
| N: Nervous system | Chlorpromazine |
|  | Levomepromazine |
|  | Haloperidol |
|  | Pimozide |
|  | Sulpiride |
|  | Levosulpiride |
|  | Hydroxyzine |
|  | Citalopram |
|  | Escitalopram |
|  | Donepezilo |
|  | Methadone |
| P: Antiparasitic products, insectcides and repellents | Chloroquine |
|  | Pentamidine isethionate |

**Patients taking antiulcer agents without criteria for gastroprotection**

| **Group therapeutic** | **Drug** | **Except** |
| --- | --- | --- |
| A: Alimentary tract and metabolism | Drugs for acid related disorders | Drugs:   - NSAIDs |
|  |  | Health problems:   - Oesophagitis - Gastro-oesophageal reflux disease - Other diseases of oesophagus - Disorders of oesophagus in diseases classified elsewhere - Gastric ulcer - Duodenal ulcer - Peptic ulcer, site unspecified - Gastrojejunal ulcer - Gastritis and duodenitis - Functional dyspepsia - Other diseases of stomach and duodenum - Ulcerative colitis - Endocrine pancreas - Abnormal secretion of gastrin |

NSAID: Non-steroidal anti- inflammatory drug

**Other drugs not recommended for the elderly**

| **Group therapeutic** | **Drug** | **Health problems** | **Patient's risk** |
| --- | --- | --- | --- |
| ﻿Gastrointestinal drugs | Proton pump inhibitors | Treatment of uncomplicated peptic ulcer or erosive esophagitis at maximum therapeutic doses for more than 8 weeks | Risk of Clostridium difficile infection, bone mass loss and fractures |
|  | Metoclopramide | Patients with Parkinson | Risk of extrapyramidal effects and tardive dyskinesia |
|  | Clebopride | Patients with Parkinson | Risk of extrapyramidal effects and tardive dyskinesia |
|  | Metoclopramide in association | Patients with Parkinson | Risk of extrapyramidal effects and tardive dyskinesia |
|  | Contact laxatives | Chronic use | Risk of diarrhea, electrolyte imbalances and abdominal distension |
|  | Antipropulsives | Patients with diarrhoea of unknown cause | Risk of diagnostic delay, constipation with incontinence, toxic megacolon in inflammatory bowel disease and may delay healing in undiagnosed gastroenteritis |
| ﻿Hypoglycaemic | Long half-life Sulfonylureas | Patients with type 2 diabetes mellitus | Risk of hypoglycaemia |
|  | Thiazolidinediones alone or in combination | Patients with heart failure | Risk of exacerbation of heart failure |
| Antiplatelet drug and anticoagulants | Ticlopidine |  | Risk of bleeding and alteration of the blood count |
|  | Prasugrel |  | Risk of bleeding |
|  | Oral anticoagulants | Patients with chronic atrial fibrillation and treated with platelet antiaggregant | Risk of bleeding |
|  | Oral anticoagulants | Patients with vascular ischemic disease with antiplatelet therapy | Risk of bleeding |
|  | Oral anticoagulants | Treatment of more than 12 months in a first episode of uncomplicated pulmonary embolism | Risk of bleeding |
|  | Oral anticoagulants | Treatment of more than 6 months in a first episode of uncomplicated deep vein thrombosis | Risk of bleeding |
| Antihypertensives | Beta blocking agents | Patients with bradycardia | Risk of asystole and complete heart block |
|  | Beta blocking agents | Patients with type 2 diabetes mellitus with frequent episodes of hypoglycemia | Risk of masking the symptoms of hypoglycemia |
|  | ACEI or ARA-II alone or in combination | Concomitant treatment with NSAID and diuretic | Risk of renal failure, especially in dehydrated patients |
|  | Trandolapril and verapamil | Patients with heart failure grade III or IV of the NYHA | Risk of decompensation of heart failure |
|  | Verapamil | Patients with heart failure grade III or IV of the NYHA | Risk of decompensation of heart failure |
|  | Diltiazem | Patients with heart failure grade III or IV of the NYHA | Risk of decompensation of heart failure |
| Diuretics | Sulfonamides,plain | Treatment of isolated malleolar edemas without signs of heart failure, decompensation of liver cirrhosis or nephrotic syndrome | Risk of electrolyte imbalance hypokalemia and hyponatremia among others |
|  | High-ceiling diuretics and potassium-sparing agents | Treatment of isolated malleolar edemas without signs of heart failure, decompensation of liver cirrhosis or nephrotic syndrome | Risk of electrolyte imbalance hypokalemia and hyponatremia among others |
|  | Sulfonamides, plain | Patients with hypertension in monotherapy | Risk of electrolyte imbalance hypokalemia and hyponatremia among others |
|  | High-ceiling diuretics and potassium-sparing agents | Patients with hypertension in monotherapy | Risk of electrolyte imbalance hypokalemia and hyponatremia among others |
| Other cardiac preparations | Trimetazidine | Except treatment for stable angina | Risk of neurological symptoms: parkinsonism, restless legs syndrome, tremor or gait instability |
|  | Ivabradine | Except intolerance or contraindication of other therapeutic alternatives | Risk of occurrence of adverse effects: digestive, neuropsychiatric and cardiovascular |
|  | Ranolazine | Except intolerance or contraindication of other therapeutic alternatives | Risk of occurrence of adverse effects: digestive, neuropsychiatric and cardiovascular |
| Genito urinary system and sex hormones | Androgens | Use in indications other than primary or secondary hypogonadism | Risk of androgenic toxicity |
|  | Estrogens | Previous history of breast cancer or venous thromboembolism. | Risk of recurrence of breast cancer or venous thromboembolism |
|  | Estrogens | Women with or without concomitant treatment with progestins | Risk of breast or endometrial cancer |
| Glucocorticoids for sistemic use | Glucocorticoids | Use over 3 months as monotherapy in rheumatoid arthritis | Risk of occurrence of adverse effects associated with corticosteroids (Cushing's syndrome, immunosuppression, osteoporosis, diabetes, cardiovascular effects) |
|  | Glucocorticoids | Maintenance treatment in moderate-severe COPD | Risk of occurrence of adverse effects associated with corticosteroids (Cushing's syndrome, immunosuppression, osteoporosis, diabetes, cardiovascular effects) |
| Antiinfectives for systemic use | Sulfamethoxazole and trimethoprim | Treatment associated with ACEI or ARA-II | Risk of sudden death |
|  | Nitrofurantoin | Use greater than 7 days | Risk of pulmonary toxicity and liver damage |
| ﻿Analgesics | Coxibs | Patients with concurrent cardiovascular disease | Risk of acute myocardial infarction and stroke |
|  | NSAIDs | Patients with severe hypertension | Risk of increase in blood pressure |
|  | NSAIDs | Patients with severe heart failure | Risk of decompensation of heart failure |
|  | NSAIDs | Use over 3 months in the chronic treatment of gout without contraindication for inhibitors of xanthine oxidase | Risk of gastrointestinal bleeding |
|  | NSAIDs | Use over 3 months, in joint pain without previous treatment with paracetamol | Risk of gastrointestinal bleeding |
|  | Indometacin |  | Risk of adverse effects at severe CNS level |
|  | Opioids | Regular treatment with opioids (except on demand) without associated laxative treatment | Constipation risk |
| Anti-dementia drugs | Anticholinesterases | Patients treated with drugs that decrease the heart rate | Risk of cardiac conduction failure, syncope or cardiac injury |
|  | Anticholinesterases | Patients with persistent bradycardia, heart block or recurrent syncope | Risk of cardiac conduction failure, syncope or cardiac injury |
| Anti-Parkinson drugs | Dopa and dopa derivates | Patients with benign essential tremor | There is no evidence of its effectiveness |
|  | Dopamine agonists | Patients with benign essential tremor | There is no evidence of its effectiveness |
| ﻿Antidepressants | Selective serotonin reuptake inhibitors | Patients with non-iatrogenic hyponatremia | Risk of severe hyponatremia |
|  | Agomelatine | Age > 75 years | Risk of hepatic toxicity |
| Psycholeptics | Neuroleptics not recommended in Parkinson | Use greater than one month in patients with Parkinson | Risk of aggravation of extrapyramidal symptoms |
|  | Antipsychotics | Patients with dementia and/or delirium | Risk of stroke and mortality |
| Others | Barbiturates and derivatives |  | Risk of dependence, tolerance and overdose. Risk of delirium or confusional states |
|  | Colchicine | Treatment of gout for a period exceeding 3 months, where there is no contraindication to a xanthine-oxidase inhibitor | Risk of toxicity: nausea, vomiting, diarrhoea and abdominal pain |
|  | Ergot alkaloids |  | Risk of peripheral vasoconstriction and cardiac ischemia |
|  | Desmopressin | Patients with nycturia | Risk of hyponatremia |
|  | Megestrol |  | Risk of thrombotic events and increased mortality |
|  | Xanthines | Patients with COPD treated with xanthines in monotherapy | Risk of adverse effects such as vomiting, agitation, dilatation of the pupils and tachycardia, among others. |

PPI: Proton pump inhibitors; CNS: Central nervous system; NSAID: Non-steroidal anti- inflammatory drug; ACEI: Angiotensin-converting-enzyme inhibitor; ARA-II: Angiotensin II receptor antagonists; COPD: Chronic obstructive pulmonary disease

**Patients needing gastroprotection**

| **Group therapeutic** | **Drug** | **Health problems** | **Patient's risk** |
| --- | --- | --- | --- |
| Salicylic acid and derivatives | Acetylsalicylic acid | Clinical history of peptic ulcer without concomitant treatment with PPI | Risk of recurrent peptic ulcer and/or digestive haemorrhage |
| Analgesics and antiinflammatory | NSAIDs | Without concomitant treatment with PPI | Risk of gastrointestinal bleeding |

PPI: Proton pump inhibitors; NSAID: Non-steroidal anti- inflammatory drug;

†References

1. Delgado Silveira E, Montero Errasquín B, Muñoz García M, Vélez-Díaz-Pallarés M, Lozano Montoya I, Sánchez-Castellano C, et al. Mejorando la prescripción de medicamentos en las personas mayores: Una nueva edición de los criterios STOPP-START. Rev Esp Geriatr Gerontol. 2015;50(2):89–96.
2. Samuel MJ. American Geriatrics Society 2015 updated beers criteria for potentially inappropriate medication use in older adults. J Am Geriatr Soc. 2015;63(11):2227–46.
3. Stefanie Holt, Sven Schmiedl PAT. Potentially Inappropriate Medications in the Elderly: The PRISCUS List. Dtsch Arztebl Int. 2010;107(31–32):543–51.
4. Laroche ML, Charmes JP, Merle L. Potentially inappropriate medications in the elderly: A French consensus panel list. Eur J Clin Pharmacol. 2007;63(8):725–31.
5. Matanović SM, Vlahovic-Palcevski V. Potentially inappropriate medications in the elderly: A comprehensive protocol. Eur J Clin Pharmacol. 2012;68(8):1123–38.
6. Ferrandis Tebar V, Moreno Sánchez E, Obreo Pintos J, Viñuela Álvarez D. Revisión de la medicación en el paciente anciano. Listado de medicamentos susceptibles de ser inadecuados. 2018.
7. Holt S, Schmiedl S, Thürmann PA. Potentially Inappropriate Medications in the Elderly: The PRISCUS List. Dtsch Arztebl Int. 2010; 107 (31–32):543–51.
8. Vanesa Ferrandis Tebar, Elena Moreno Sánchez, Juana Obreo Pintos, Diana Viñuela Álvarez. Revisión de la medicación en el paciente anciano. Listado de medicamentos susceptibles de ser inadecuados. Sacyl. 2017.
9. Agencia Española del Medicamento y Productos Sanitarios. Nota informativa 19/07/2016: Trimetazidina: se recuerda que actualmente su única indicación autorizada es el tratamiento de la angina de pecho.
10. Agencia Española del Medicamento y Productos Sanitarios. Nota informativa 22/3/2013: Cilostazol (Ekistol, Pletal): finalización de la revaluación de la relación beneficio - riesgo y restricciones de uso.
11. Agencia Española del Medicamento y Productos Sanitarios. Nota informativa 13/02/2015: Hidroxizina (Atarax®): nuevas restricciones de uso para minimizar su riesgo arritmogénico.
12. Fralick M, Macdonald EM, Gomes T, Antoniou T, Hollands S, Mamdani MM, Juurlink DN; Canadian Drug Safety and Effectiveness Research Network (CDSERN). Co-trimoxazole and sudden death in patients receiving inhibitors of renin-angiotensin system: population based study. BMJ 2014;349:g6196.
13. Francis J, Young GB. Diagnosis of delirium and conusional states. [Monografía en Internet]. Walthman (MA): UpToDate; 2018 [acceso 10 de febrero de 2018]. Disponible en: <http://www.uptodate.com/>
14. Chew ML, Mulsant BH, Pollock BG, Lehman ME, Greenspan A, Mahmoud RA, et al. Anticholinergic Activity of 107 Medications Commonly Used by Older Adults. J Am Geriatr Soc 2008;56:1333–1341.
15. Bonafont X, Llop R. Medicaments i caigudes. BIT 2017;28:28-33.
16. Pour mieux soigner, des médicaments à écarter : bilan 2018. Rev Prescrire. 2018; 38 (412): 135-14.
17. Durán CE, Azermai M, Vander Stichele RH. Systematic review of anticholinergic risk scales in older adults. Eur J Clin Pharmacol. 2013; 69(7):1485-96.
18. Rochon PA. Drug prescribing for older adults. Schmader KE, Givens J, ed.: Uptodate; 2020. [cited 2019 Nov 30]. Available from: <http://www.uptodate.com/>
19. Woosley RL, Heise CW , Gallo T, Tate J, Woosley D and Romero KA, [www.CredibleMeds.org](http://www.CredibleMeds.org), QTdrugs List, [cited 2019 Nov 30], AZCERT, Inc. 1822 Innovation Park Dr., Oro Valley, AZ 85755

**Table S8. Number of contraindicated drugs in chronic kidney disease and liver disease and potentially inappropriate medications in older people by multimorbidity group (853,085 older people, Catalonia, 2012).**

| Medication-related problem | Number of drugs^†^ | **Multimorbidity**  (2-4 diseases)  (n=268,836) | **Multimorbidity**  (5-9 diseases)  (n=463,709) | **Multimorbidity**  (≥10 diseases)  (n=120,540) | **All**  (N=853,085) |
| --- | --- | --- | --- | --- | --- |
| Contraindicated drugs in chronic kidney disease,  n (%) | 0 | 260,488 (96.9) | 399,243 (86.1) | 82,830 (68.7) | 742,561 (87.0) |
|  | 1 | 5,690 (2.12) | 33,116 (7.14) | 14,951 (12.4) | 53,757 (6.30) |
|  | 2 | 1,983 (0.74) | 19,497 (4.20) | 12,300 (10.2) | 33,780 (3.96) |
|  | 3 | 542 (0.20) | 8,366 (1.80) | 6,633 (5.50) | 15,541 (1.82) |
|  | 4 | 114 (0.04) | 2,685 (0.58) | 2,700 (2.24) | 5,499 (0.64) |
|  | 5 | 14 (0.01) | 623 (0.13) | 863 (0.72) | 1,500 (0.18) |
|  | 6 | 3 (0.00) | 140 (0.03) | 212 (0.18) | 355 (0.04) |
|  | 7 | 2 (0.00) | 33 (0.01) | 38 (0.03) | 73 (0.01) |
|  | 8 | 0 (0.00) | 4 (0.00) | 8 (0.01) | 12 (0.00) |
|  | 9 | 0 (0.00) | 2 (0.00) | 4 (0.00) | 6 (0.00) |
|  | 10 | 0 (0.00) | 0 (0.00) | 1 (0.00) | 1 (0.00) |
| Contraindicated drugs in liver disease, n (%) | 0 | 263,288 (97.9) | 441,995 (95.3) | 109,897 (91.2) | 815,180 (95.6) |
|  | 1 | 3,293 (1.22) | 10,111 (2.18) | 3,762 (3.12) | 17,166 (2.01) |
|  | 2 | 1,629 (0.61) | 6,515 (1.40) | 3,029 (2.51) | 11,173 (1.31) |
|  | 3 | 469 (0.17) | 3,480 (0.75) | 2,247 (1.86) | 6,196 (0.73) |
|  | 4 | 131 (0.05) | 1,182 (0.25) | 1,097 (0.91) | 2,410 (0.28) |
|  | 5 | 20 (0.01) | 322 (0.07) | 392 (0.33) | 734 (0.09) |
|  | 6 | 6 (0.00) | 81 (0.02) | 90 (0.07) | 177 (0.02) |
|  | 7 | 0 (0.00) | 18 (0.00) | 23 (0.02) | 41 (0.00) |
|  | 8 | 0 (0.00) | 4 (0.00) | 3 (0.00) | 7 (0.00) |
|  | 9 | 0 (0.00) | 1 (0.00) | 0 (0.00) | 1 (0.00) |
| Potentially inappropriate medications, n (%) | 0 | 143,989 (53.6) | 129,787 (28.0) | 14,420 (12.0) | 288,196 (33.8) |
|  | 1 | 62,756 (23.3) | 117,545 (25.3) | 21,036 (17.5) | 201,337 (23.6) |
|  | 2 | 30,643 (11.4) | 83,771 (18.1) | 22,761 (18.9) | 137,175 (16.1) |
|  | 3 | 15,271 (5.68) | 53,520 (11.5) | 18,986 (15.8) | 87,777 (10.3) |
|  | 4 | 7,777 (2.89) | 32,586 (7.03) | 14,282 (11.8) | 54,645 (6.41) |
|  | 5 | 3,955 (1.47) | 19,674 (4.24) | 10,363 (8.60) | 33,992 (3.98) |
|  | 6 | 2,004 (0.75) | 11,445 (2.47) | 7,062 (5.86) | 20,511 (2.40) |
|  | 7 | 1,128 (0.42) | 6,677 (1.44) | 4,534 (3.76) | 12,339 (1.45) |
|  | 8 | 629 (0.23) | 3,830 (0.83) | 2,774 (2.30) | 7,233 (0.85) |
|  | 9 | 311 (0.12) | 2,209 (0.48) | 1,774 (1.47) | 4,294 (0.50) |
|  | ≥10 | 373 (0.14) | 2,665 (0.57) | 2,548 (2.11) | 5,586 (0.65) |

^†^Number of Anatomical Therapeutic Chemical (ATC) at 5^th^ level.

Note: All variables of the table showed a significant difference (p<0.001) between multimorbidity groups.

**Table S9. Twenty most contraindicated drugs in chronic kidney disease by multimorbidity group (853,085 older people, Catalonia, 2012).**

| **Code of ATC (drug),**  **n (%)** | **Multimorbidity**  (2-4 diseases)  (n=268,836) | **Multimorbidity**  (5-9 diseases)  (n=463,709) | **Multimorbidity**  (≥10 diseases)  (n=120,540) | **All**  (N=853,085) |
| --- | --- | --- | --- | --- |
| A10BA02 (Metformin) | 731 (0.27) | 13,655 (2.94) | 10,465 (8.68) | 24,851 (2.91) |
| C03AA03 (Hydrochlorothiazide) | 1,530 (0.57) | 11,043 (2.38) | 5,488 (4.55) | 18,061 (2.12) |
| C09BA02 (Enalapril and diuretics) | 1,238 (0.46) | 7,981 (1.72) | 3,418 (2.84) | 12,637 (1.48) |
| N06AB04 (Citalopram) | 321 (0.12) | 4,483 (0.97) | 4,639 (3.85) | 9,443 (1.11) |
| M01AE01 (Ibuprofen) | 579 (0.22) | 4,495 (0.97) | 2,620 (2.17) | 7,694 (0.90) |
| M05BA04 (Alendronic acid) | 234 (0.09) | 3,828 (0.83) | 3,518 (2.92) | 7,580 (0.89) |
| A10BB09 (Gliclazide) | 185 (0.07) | 3,546 (0.76) | 2,451 (2.03) | 6,182 (0.72) |
| C09DA01 (Losartan and diuretics) | 421 (0.16) | 3,505 (0.76) | 1,952 (1.62) | 5,878 (0.69) |
| C09CA03 (Valsartan) | 407 (0.15) | 3,286 (0.71) | 2,129 (1.77) | 5,822 (0.68) |
| N02AX02 (Tramadol) | 166 (0.06) | 2,617 (0.56) | 2,964 (2.46) | 5,747 (0.67) |
| C09DA03 (Valsartan and diuretics) | 462 (0.17) | 3,289 (0.71) | 1,665 (1.38) | 5,416 (0.63) |
| C03DA01 (Spironolactone) | 140 (0.05) | 2,046 (0.44) | 2,825 (2.34) | 5,011 (0.59) |
| C01EB15 (Trimetazidine) | 284 (0.11) | 2,246 (0.48) | 1,499 (1.24) | 4,029 (0.47) |
| M01AB05 (Diclofenac) | 229 (0.09) | 2,089 (0.45) | 1,264 (1.05) | 3,582 (0.42) |
| A10BB01 (Glibenclamide) | 131 (0.05) | 2,032 (0.44) | 1,369 (1.14) | 3,532 (0.41) |
| C08CA11 (Manidipine) | 212 (0.08) | 1,972 (0.43) | 1,297 (1.08) | 3,481 (0.41) |
| C09CA08 (Olmesartan medoxomil) | 249 (0.09) | 1,658 (0.36) | 945 (0.78) | 2,852 (0.33) |
| C09BA03 (Lisinopril and diuretics) | 286 (0.11) | 1,772 (0.38) | 774 (0.64) | 2,832 (0.33) |
| C10AB05 (Fenofibrate) | 160 (0.06) | 1,577 (0.34) | 1,077 (0.89) | 2,814 (0.33) |
| A12BA04 (Potassium hydrogencarbonate) | 83 (0.03) | 1,192 (0.26) | 1,444 (1.20) | 2,719 (0.32) |

**Table S10. Twenty most contraindicated drugs in liver disease by multimorbidity group (853,085 older people, Catalonia, 2012).**

| **Code of ATC (drug),**  **n (%)** | **Multimorbidity**  (2-4 diseases)  (n=268,836) | **Multimorbidity**  (5-9 diseases)  (n=463,709) | **Multimorbidity**  (≥10 diseases)  (n=120,540) | **All**  (N=853,085) |
| --- | --- | --- | --- | --- |
| C10AA01 (Simvastatin) | 2,077 (0.77) | 8,274 (1.78) | 4,018 (3.33) | 14,369 (1.68) |
| A10BA02 (Metformin) | 748 (0.28) | 4,383 (0.95) | 2,421 (2.01) | 7,552 (0.89) |
| C03CA01 (Furosemide) | 275 (0.10) | 3,066 (0.66) | 3,252 (2.70) | 6,593 (0.77) |
| C10AA05 (Atorvastatin) | 736 (0.27) | 3,490 (0.75) | 2,134 (1.77) | 6,360 (0.75) |
| C03AA03 (Hydrochlorothiazide) | 641 (0.24) | 2,475 (0.53) | 1,039 (0.86) | 4,155 (0.49) |
| C09BA02 (Enalapril and diuretics) | 523 (0.19) | 1,718 (0.37) | 645 (0.54) | 2,886 (0.34) |
| N05CD06 (Lormetazepam) | 283 (0.11) | 1,394 (0.30) | 943 (0.78) | 2,620 (0.31) |
| C09CA01 (Losartan) | 232 (0.09) | 1,104 (0.24) | 802 (0.67) | 2,138 (0.25) |
| C10AA03 (Pravastatin) | 173 (0.06) | 1,070 (0.23) | 757 (0.63) | 2,000 (0.23) |
| C03CA04 (Torasemide) | 168 (0.06) | 1,021 (0.22) | 754 (0.63) | 1,943 (0.23) |
| C09CA03 (Valsartan) | 139 (0.05) | 701 (0.15) | 446 (0.37) | 1,286 (0.15) |
| C09DA01 (Losartan and diuretics) | 147 (0.05) | 729 (0.16) | 348 (0.29) | 1,224 (0.14) |
| C09DA03 (Valsartan and diuretics) | 186 (0.07) | 676 (0.15) | 282 (0.23) | 1,144 (0.13) |
| N05CF02 (Zolpidem) | 111 (0.04) | 496 (0.11) | 314 (0.26) | 921 (0.11) |
| A10BX02 (Repaglinide) | 42 (0.02) | 438 (0.09) | 396 (0.33) | 876 (0.10) |
| C10AB05 (Fenofibrate) | 101 (0.04) | 425 (0.09) | 237 (0.20) | 763 (0.09) |
| C10AA07 (Rosuvastatin) | 110 (0.04) | 387 (0.08) | 209 (0.17) | 706 (0.08) |
| C09CA06 (Candesartan) | 90 (0.03) | 362 (0.08) | 225 (0.19) | 677 (0.08) |
| C10AB04 (Genfibrozil) | 87 (0.03) | 390 (0.08) | 197 (0.16) | 674 (0.08) |
| A05AA02 (Ursodeoxycholic acid) | 105 (0.04) | 375 (0.08) | 170 (0.14) | 650 (0.08) |

**Table S11. Twenty most potentially inappropriate medications in older people by multimorbidity group (853,085 older people, Catalonia, 2012).**

| **Code of ATC (drug),**  **n (%)** | **Multimorbidity**  (2-4 diseases)  (n=268,836) | **Multimorbidity**  (5-9 diseases)  (n=463,709) | **Multimorbidity**  (≥10 diseases)  (n=120,540) | **All**  (N=853,085) |
| --- | --- | --- | --- | --- |
| A02BC01 (Omeprazole) | 35,974 (13.38) | 121,457 (26.19) | 44,797 (37.16) | 202,228 (23.71) |
| N05BA06 (Lorazepam) | 21,295 (7.92) | 62,587 (13.50) | 23,655 (19.62) | 107,537 (12.61) |
| G04CA02 (Tamsulosin) | 10,584 (3.94) | 28,700 (6.19) | 9,191 (7.62) | 48,475 (5.68) |
| N05CD06 (Lormetazepam) | 8,754 (3.26) | 27,497 (5.93) | 10,931 (9.07) | 47,182 (5.53) |
| N05BA12 (Alprazolam) | 7,877 (2.93) | 23,206 (5.00) | 8,942 (7.42) | 40,025 (4.69) |
| N06AB04 (Citalopram) | 5,058 (1.88) | 22,483 (4.85) | 11,533 (9.57) | 39,074 (4.58) |
| M01AE01 (Ibuprofen) | 6,719 (2.50) | 21,808 (4.70) | 7,190 (5.96) | 35,717 (4.19) |
| N06AB05 (Paroxetine) | 4,468 (1.66) | 16,256 (3.51) | 6,712 (5.57) | 27,436 (3.22) |
| C02CA04 (Doxazosin) | 4,439 (1.65) | 15,241 (3.29) | 6,289 (5.22) | 25,969 (3.04) |
| N02AX52 (Tramadol, combinations) | 3,528 (1.31) | 14,701 (3.17) | 6,822 (5.66) | 25,051 (2.94) |
| N02AX02 (Tramadol) | 2,545 (0.95) | 13,281 (2.86) | 7,589 (6.30) | 23,415 (2.74) |
| N05BA01 (Diazepam) | 3,999 (1.49) | 11,956 (2.58) | 4,725 (3.92) | 20,680 (2.42) |
| N06AB06 (Sertraline) | 2,668 (0.99) | 11,839 (2.55) | 6,159 (5.11) | 20,666 (2.42) |
| A02BC02 (Pantoprazole) | 2,987 (1.11) | 11,499 (2.48) | 5,722 (4.75) | 20,208 (2.37) |
| C01EB15 (Trimetazidine) | 4,208 (1.57) | 11,248 (2.43) | 3,810 (3.16) | 19,266 (2.26) |
| A02BA02 (Ranitidine) | 3,095 (1.15) | 10,540 (2.27) | 4,418 (3.67) | 18,053 (2.12) |
| N06AB10 (Escitalopram) | 2,958 (1.10) | 10,124 (2.18) | 4,496 (3.73) | 17,578 (2.06) |
| N05CF02 (Zolpidem) | 3,159 (1.18) | 9,583 (2.07) | 3,966 (3.29) | 16,708 (1.96) |
| A10BB01 (Glibenclamide) | 2,609 (0.97) | 10,247 (2.21) | 3,479 (2.89) | 16,335 (1.91) |
| G03CA09 (Promestriene) | 3,419 (1.27) | 9,321 (2.01) | 3,338 (2.77) | 16,078 (1.88) |
